# Supplementary material for: Child and caregiver mental health during 12 months of the COVID-19 pandemic in Australia: findings from national repeated cross-sectional surveys
Source: BMJ Paediatr Open. 2022 Jul 28;6(1):e001390. doi: 10.1136/bmjpo-2021-001390 (PMC9341184; doi:10.1136/bmjpo-2021-001390)
Supplement: Supplementary data [file bmjpo-2021-001390supp001.pdf]

Supplementary Table 1: Estimated probabilities over time (by survey) and by state/territory (as a proxy for total length of lockdown) for the three mental health measures (graphed in Figure 1)

A. Poor caregiver mental health (Kessler-6)

	**Proportion[a, b]**	**Risk Difference[a, b]**	**95% Confidence Interval[a, b]**		**p-values[a, b]**
			Lower	Upper	
**Other States + W1 June 2020**	0.12				
**Other States + W2 Sep 2020**	0.17	0.05	-0.01	0.10	0.093
**Other States + W3 July 2021**	0.13	0.01	-0.02	0.04	0.589
**NSW + W1 June 2020**	0.19	0.07	0.02	0.12	0.005
**NSW + W2 Sep 2020**	0.17	0.05	0.01	0.10	0.024
**NSW + W3 July 2021**	0.23	0.11	0.06	0.15	0.000
**VIC + W1 June 2020**	0.17	0.05	0.00	0.10	0.033
**VIC + W2 Sep 2020**	0.25	0.13	0.07	0.18	0.000
**VIC + W3 July 2021**	0.26	0.14	0.09	0.18	0.000

[a] All estimates are adjusted for caregiver's gender, family structure (sole-caregiving), parent's education, Socio-Economic Indexes for Areas Index of Relative Disadvantage (SEIFA) score, and cultural and linguistic diversity as well as weighted using national demographic distributions for caregiver age, gender, family structure (sole-caregiving, number of children and any under 5 years), state/territory and SEIFA.
[b] Generalised linear model of the binomial family was used for analysis.

B. Perceived negative impacts of the pandemic on caregiver mental health

	**Proportion**[a, b]	**Risk Difference**[a, b]	**95% Confidence Interval**[a, b]		**p-values**[a, b]
			Lower	Upper	
**Other States + W1 June 2020**	0.44				
**Other States + W2 Sep 2020**	0.44	0.00	-0.09	0.08	0.912
**Other States + W3 July 2021**	0.57	0.12	0.05	0.19	0.000
**NSW + W1 June 2020**	0.50	0.06	-0.02	0.14	0.132
**NSW + W2 Sep 2020**	0.49	0.05	-0.03	0.13	0.224
**NSW + W3 July 2021**	0.61	0.16	0.09	0.23	0.000
**VIC + W1 June 2020**	0.50	0.06	-0.02	0.14	0.130
**VIC + W2 Sep 2020**	0.57	0.13	0.05	0.21	0.001
**VIC + W3 July 2021**	0.67	0.23	0.16	0.30	0.000

[a] All estimates are adjusted for caregiver's gender, family structure (sole-caregiving), parent's education, Socio-Economic Indexes for Areas Index of Relative Disadvantage (SEIFA) score, and cultural and linguistic diversity as well as weighted using national demographic distributions for caregiver age, gender, family structure (sole-caregiving, number of children and any under 5 years), state/territory and SEIFA.
[b] Generalised linear model of the binomial family was used for analysis.

C. Perceived negative impacts of the pandemic on children's mental health

	**Proportion[a, b]**	**Risk Difference[a, b]**	**95% Confidence Interval[a, b]**		**p-values[a, b]**
			Lower	Upper	
**Other States + W1 June 2020**	0.27				
**Other States + W2 Sep 2020**	0.21	-0.07	-0.13	-0.00	0.046
**Other States + W3 July 2021**	0.35	0.08	0.02	0.14	0.014
**NSW + W1 June 2020**	0.27	-0.01	-0.08	0.06	0.826
**NSW + W2 Sep 2020**	0.35	0.08	0.00	0.15	0.049
**NSW + W3 July 2021**	0.48	0.21	0.15	0.28	0.000
**VIC + W1 June 2020**	0.25	-0.02	-0.08	0.04	0.527
**VIC + W2 Sep 2020**	0.42	0.15	0.07	0.22	0.000
**VIC + W3 July 2021**	0.51	0.24	0.18	0.30	0.000

[a] All estimates are adjusted for caregiver's gender, family structure (sole-caregiving), parent's education, Socio-Economic Indexes for Areas Index of Relative Disadvantage (SEIFA) score, cultural and linguistic diversity, parent's mental health (Kessler-6) and clustering at the level of family as well as weighted using national demographic distributions for caregiver age, gender, family structure (sole-caregiving, number of children and any under 5 years), state/territory and SEIFA.
[b] Generalised linear model of the binomial family was used for analysis.
