# Supplementary material for: Child and caregiver mental health during 12 months of the COVID-19 pandemic in Australia: findings from national repeated cross-sectional surveys
Source: BMJ Paediatr Open. 2022 Jul 28;6(1):e001390. doi: 10.1136/bmjpo-2021-001390 (PMC9341184; doi:10.1136/bmjpo-2021-001390)
Supplement: Author's manuscript [file bmjpo-2021-001390.draft_revisions.pdf]

BMJ Paediatrics Open

BMJ Paediatrics Open is committed to open peer review. As part of this commitment we make the peer review history of every article we publish publicly available.

When an article is published we post the peer reviewers' comments and the authors' responses online. We also post the versions of the paper that were used during peer review. These are the versions that the peer review comments apply to.

The versions of the paper that follow are the versions that were submitted during the peer review process. They are not the versions of record or the final published versions. They should not be cited or distributed as the published version of this manuscript.

BMJ Paediatrics Open is an open access journal and the full, final, typeset and author-corrected version of record of the manuscript is available on our site with no access controls, subscription charges or pay-per-view fees (http://bmjpaedsopen.bmj.com).

If you have any questions on BMJ Paediatrics Open's open peer review process please email info.bmjpo@bmj.com

BMJ Paediatrics Open

Child and caregiver mental health during 12 months of the COVID-19 pandemic in Australia

Journal:	*BMJ Paediatrics Open*
Manuscript ID	bmjpo-2021-001390
Article Type:	Original research
Date Submitted by the Author:	24-Jan-2022

Complete List of Authors:	Price, Anna; Murdoch Childrens Research Institute, Centre for Community Child Health; The University of Melbourne, Paediatrics<br>Measey, Mary-Anne; The Royal Children's Hospital Melbourne, General Medicine<br>Hoq, Monsurul; Murdoch Childrens Research Institute, Clinical Epidemiology and Biostatistics Unit<br>Rhodes, Anthea; Royal Children's Hospital Melbourne, General Medicine<br>Goldfeld, Sharon; Royal Children's Hospital Melbourne, Centre for Community Child Health

Keywords:	COVID-19, Psychology

SCHOLARONE™
Manuscripts

I, the Submitting Author has the right to grant and does grant on behalf of all authors of the Work (as defined in the below author licence), an exclusive licence and/or a non-exclusive licence for contributions from authors who are: i) UK Crown employees; ii) where BMJ has agreed a CC-BY licence shall apply, and/or iii) in accordance with the terms applicable for US Federal Government officers or employees acting as part of their official duties; on a worldwide, perpetual, irrevocable, royalty-free basis to BMJ Publishing Group Ltd ("BMJ") its licensees and where the relevant Journal is co-owned by BMJ to the co-owners of the Journal, to publish the Work in this journal and any other BMJ products and to exploit all rights, as set out in our licence.

The Submitting Author accepts and understands that any supply made under these terms is made by BMJ to the Submitting Author unless you are acting as an employee on behalf of your employer or a postgraduate student of an affiliated institution which is paying any applicable article publishing charge ("APC") for Open Access articles. Where the Submitting Author wishes to make the Work available on an Open Access basis (and intends to pay the relevant APC), the terms of reuse of such Open Access shall be governed by a Creative Commons licence – details of these licences and which Creative Commons licence will apply to this Work are set out in our licence referred to above.

Other than as permitted in any relevant BMJ Author's Self Archiving Policies, I confirm this Work has not been accepted for publication elsewhere, is not being considered for publication elsewhere and does not duplicate material already published. I confirm all authors consent to publication of this Work and authorise the granting of this licence.

Confidential: For Review Only

Child and caregiver mental health during 12 months of the COVID-19 pandemic in Australia

Authors:
*Anna M. H. Price[1,2,3], Mary-Anne Measey[2, 4], Monsurul Hoq[5], Anthea Rhodes[2,3,6, ¶], Sharon Goldfeld[1,2,3,¶]

Affiliations^:
1. Centre for Community Child Health, The Royal Children's Hospital, Parkville, Vic
2. Population Health, Murdoch Children's Research Institute, Parkville, Vic
3. Department of Paediatrics, The University of Melbourne, Parkville, Vic
4. Health Services Research Unit, The Royal Children's Hospital, Parkville, Vic
5. Clinical Epidemiology and Biostatistics Unit, Murdoch children's Research Institute, Parkville, Vic
6. Department of General Medicine, The Royal Children's Hospital, Parkville, Vic

^All Australia,
*Corresponding author, anna.price@mcri.edu.au
¶ AR and SG are joint senior authors.

Word Count: 2577 words

Confidential: For Review Only

ABSTRACT

Background: There are urgent calls for research into the mental health consequences of living through the COVID-19 pandemic. Australia's effective suppression of COVID-19 offers insights into the pandemic mental health impacts in the relative absence of the disease. We aimed to describe the mental health experiences of caregivers and children over 12 months, reporting differences related to demographic, socioeconomic and lockdown characteristics.

Methods: Data were drawn from Australia's only nationally representative, repeated cross-sectional survey of caregivers with children (0-17 years). N=2020 caregivers participated in June 2020, N=1434 in September 2020, and N=2508 in July 2021. Caregivers reported their mental health (poor versus not, Kessler-6), and mental health impacts of the pandemic (negative versus none/positive) for themselves and their children. Data were weighted to approximate population distributions of caregiver age, gender, sole-caregiving, number and ages of children, state/territory and neighbourhood-level disadvantage.

Results: Negative mental health impacts were more common for female (vs male) caregivers, older (vs younger) children. Poor caregiver mental health (K6) was more common for families experiencing socioeconomic adversity (especially financial), while perceived negative impacts were more common for more socially advantaged groups. Caregivers who experienced the least total lockdown reported similar K6 over time. Otherwise, poor mental health and negative mental health impacts increased over time with cumulative lockdown.

Conclusion: Despite Australia's low infection rates, the mental health impacts of the pandemic and related public health restrictions are real and concerning. Addressing poor mental health must be central to ongoing pandemic recovery efforts for families and children.

What is known about this topic?

- The global evidence shows that, for general adult populations, psychological distress peaked in the first months of the COVID-19 pandemic before appearing to improve.
- Less is known about mental health over time of living through the pandemic, especially for caregivers and children. There are urgent calls for research.
- Due to low infection rates, Australia's experience can provide insight into the mental health impacts of lockdown with minimal compounding harms of the virus.

What this study adds:

- From June 2020 to July 2021, Australia's lockdowns were detrimental for caregiver and child mental health.
- There was an unequal distribution of mental health impacts based on caregiver gender, child age and family socioeconomic characteristics.
- Pandemic response and recovery planning must consider both family mental health and socioeconomic security.

Confidential: For Review Only

INTRODUCTION

During the first eighteen months of the COVID-19 pandemic, balancing the direct harms of the virus with the indirect harms of public health restrictions was a global challenge. Australia enacted some of the strictest public health measures in the world to effectively suppress infection.[1] By 31 July 2021, the measures successfully contained infection to an overall incidence rate of 137 cases and 3.7 deaths per 100,000 people.[2] With just 15% of the population of 25 million fully vaccinated by the end of July 2021, stay-at-home orders ('lockdown') were the country's main measure of disease control.[3] Families caring for children and young people (referred to as children hereafter) have been substantially affected by lockdown,[4 5] required to juggle working from home, responsibility for their children's remote learning and care during school and childcare closures, with minimal social opportunities due to closure of sports and play centres, and reduced access to healthcare. Despite mental health having "greater impact on human activity than any other non-communicable illness",[4] it appeared to be given little consideration in the majority of Australia's lockdown policies. With future variants and pandemics likely, Australia's experience offers insight into the mental health impacts of lockdown, effectively without the compounding direct harms of the virus.

The evidence for the indirect and longer-term impacts of the pandemic is quickly evolving. In a review of the global mental health evidence from the first year (to April 2021), Aknin et al., reported a peak in adults' psychological distress in the early months.[4] While many studies reported a decline to pre-pandemic levels by mid-2020,[4] the authors found that mental health inequities were sustained or exacerbated for adults who were younger, female, child-rearing, or with fewer socioeconomic resources.[4 5] However, published data on the specific experience of parents (termed caregivers throughout) are scarce. Evidence reviews of children's experiences also suggest that their mental health is declining during the pandemic.[6 7] In Racine and colleagues' (2021) meta-analysis of 29 studies published in the first year of the pandemic, prevalence estimates for depression and anxiety in

https://mc.manuscriptcentral.com/bmjpo

Confidential: For Review Only

children doubled, and were higher over time, and for older adolescents and females.[8] However, the available systematic reviews are limited by over-representation of data from the early months of the pandemic,[6-8] and previous pandemics show that negative mental health effects can persist.[9] As such, there are urgent calls for research into the "long-term mental health consequences of living through the pandemic",[4] which prioritises children.[5]

In response to these calls, we aimed to describe the impact of the pandemic on caregiver and child mental health using Australia's only nationally representative, repeated cross-sectional survey of families during the pandemic. Unlike many high-income countries, Australia's low incidence of COVID-19 makes it possible to examine the mental health experiences in the relative absence of the disease. Based on the gender, age and socioeconomic disparities identified in the published evidence reviews,[4 8] we hypothesised that poor mental health and negative impacts of the pandemic on mental health would be more common: (i) for caregivers who identified as female compared with male, and older compared with younger children, (ii) for families experiencing greater socioeconomic adversity compared with more socioeconomic advantage and (iii) with cumulative (increasing total length) of lockdown.

Confidential: For Review Only

METHODS

Design and procedure: The Royal Children's Hospital (RCH) National Child Health Poll comprises periodic cross-sectional surveys of approximately 2000 Australian caregivers of children aged 0-17 years. Data collection is contracted to the Online Research Unit who obtain written informed consent and draw a nationally representative sample of caregivers using stratified random sampling from their panel of over 350,000 adults aged 18 years or older, who live in Australia and have internet access. Surveys are administered in sixth grade-equivalent (end of elementary school) English, anonymous, and respondents are remunerated with points exchangeable for department store gift vouchers. Questions about mental health – the focus of the current study – were introduced after COVID-19 began and were collected in three surveys. The dates of data collection were: (1) 15-23 June 2020, after a first national lockdown (March-May 2020) eased; (2) 15-29 September 2020, when only metropolitan residents of Victoria were in a second, stricter lockdown (July-November 2020); and (3) 20-29 July 2021, when multiple states/territories were in and out of lockdown (June 2021 onwards)). The RCH Human Research Ethics Committee approved this research (February 2020, #35254).

Patient and Public Involvement: The research questions and design of this study were informed by previous RCH Poll surveys, which asked caregivers to identify the child health issues of most concern to them and which child health topics should be included in future polls. At the end of each survey, participants were informed of the study website where all research reports are accessible to the public. As each survey is collected from a cross-sectional, population-based online survey of a random sample, respondents were not directly involved in the recruitment or conduct of the study.

Measures: Each survey collected the demographic, socioeconomic and pandemic-related financial characteristics described in Table . Table 1 also describes how measures of current and cumulative

Confidential: For Review Only

lockdown were defined. Families were assigned the Australian Bureau of Statistics' (ABS) Socio-Economic Indexes for Areas (SEIFA) Index of Relative Disadvantage, a national area level index derived from census data for all individuals living in a postcode, with higher scores indicating greater advantage. Caregivers self-reported their mental health (Kessler-6), and the impact of the pandemic on their own and their children's mental health (details in Table 1).

Analysis: To reduce effects of non-response and non-coverage and therefore approximate the population distributions of mental health experiences, the mental health measures were weighted using ABS distributions of caregiver age, gender, family structure (sole-caregiving, number of children and any under 5 years), state/territory and SEIFA. Mental health measures were described with weighted proportions for each survey, overall and by demographic, socioeconomic and pandemic-related financial and lockdown characteristics. For each of the three mental health measures, the association between mental health measure and time of the survey, according to state/territory grouping (as a proxy for cumulative lockdown, see Table 1), were calculated using generalised linear models. The binomial models investigated whether cumulative lockdown worsened the mental health measures over time, after adjusting for caregiver gender, sole caregiver status, education, SEIFA, and cultural or linguistic diversity (measures described in Table 1). The child mental health model was additionally adjusted for poor caregiver mental health (Kessler-6, see Table 1) and clustering at the level of family. The estimated models were then used to predict probabilities of each mental health outcome, by state/territory grouping, at the three survey timepoints, adjusted for the covariates in the model.

Confidential: For Review Only

Table 1. Demographic, socioeconomic, lockdown and mental health measures

Measure	Description
Demographic	
Age	Collected for caregivers and children, reported in years. Child ages were categorised into pre-school (0-4 years), primary/elementary school (5-11 years) and high school (12-17 years).
Gender	Collected for caregivers and children: response options "male", "female", "other". Two caregivers and no children identified as "other". Before the 2020 Australian census, "other" gender data were not collected for the Australian population, so it was not possible to calculate weights for these caregivers. Hence, they were dropped from the analysis, and the gender variable was dichotomised into "female" compared with "male".
Sole caregiver	Question "Are you the sole (single) parent or carer of a child 17 years of age or younger?", binary response options "yes" (one-caregiver household) compared with "no" (multi-caregiver household).
Caregiver education	Question "What is the highest level of schooling / education you have completed?" Trichotomised into "Year 12 or less" (response options: less than year 10, Year 10 or equivalent (e.g. school certificate), Year 12 or equivalent); "vocational training certificate" (response options: trade/apprenticeship (e.g. carpenter), certificate/diploma (e.g. Cert IV Childcare)); or "university degree" (response options: undergraduate university degree, postgraduate university degree (e.g. Masters, Doctorate, PhD).
Caregiver country of birth	Question "Were you born in Australia", binary response options "yes" (born in Australia) compared with "no" (outside Australia).
Home language	Question "Do you speak a language other than English at home?", binary response options "yes" (other than English) compared with "no" (English).
Cultural or linguistic diversity	A composite of the two above variables, caregiver country of birth and home language, to represent respondents who answered "yes" to either or both items, compared with "no" (answering no to both items).
Socioeconomic and lockdown	
Job/income loss during COVID-19	Three items drawn from the CoRonavIruS Health Impact Survey (CRISIS) caregiver version:[10] "What changes in employment or income have occurred in your household due to coronavirus/COVID-19?" (binary response options "yes" compared with "no") including: "job loss by one caregiver"; "job loss by two caregivers" and "reduced total household income". A binary variable describing any job loss (by one or two caregivers) or reduction in income due to COVID-19 (compared with not) was created.
Low income (<$1000)	A binary variable based on current total household income before tax, categorized into 10 options ranging from "less than $500 p/week" to "more than $3,000 p/week", plus "prefer not to say". The binary variable summarized low income ("less than AU$1,000 p/week" compared with more) based on Australian thresholds for income-poverty.[11] n=767 caregivers preferred not to report income, so this variable should be interpreted with caution.
Couldn't afford essential items	Eight items adapted from the Household, Income and Labour Dynamics in Australia (HILDA) Survey Wave 18 Household Questionnaire Material Deprivation Module [12] asking "In the last month, because of money pressure did you miss or put off" (binary response options: "yes" compared with "no"): mortgage or rent repayments; electricity, gas, water bills; food; healthcare; prescription medicines; home or car insurance; mobile phone bills; and internet. A binary summary variable was created to denote "any material deprivation" inability to pay for one or more essential items compared with "none".
Current lockdown	All Australian states and territories experienced a first national, 10-week lockdown from 23 March to 1 June 2020. Victoria experienced a second extended and more severe, 20-week lockdown from 8 July-23 November 2020. There were several short (less than a week) lockdowns in the intervening months across states, before New South Wales experienced its second extended period of lockdown, which began incrementally on 26 June 2021. Victoria experienced its fifth (in total) lockdown from 16-27 July 2021, before an extended lockdown subsequently began a week later on 5 August 2021. Current lockdown was therefore categorised as follows.

Confidential: For Review Only

	• The national lockdown had eased for the collection of the June 2020 Poll, so no (denoted 'N' in Tables 2-3) Australians were in lockdown. Thus, the number/proportion not in lockdown represents the whole cohort (and is equal to the 'overall' numbers/proportions at top of Tables 2-3).<br>• For the September 2020 Poll, only residents of metropolitan Melbourne in the state of Victoria were in lockdown (denoted 'Y' for "yes") compared with all other Australians (N). This was categorised according to respondents who reported living in Victoria and living in "metropolitan" compared with "regional/rural" areas.<br>• By July 2021, many states/territories were going in and out of lockdown, and this question was added into the Poll and reported on directly with the question "are you currently under stay-at-home orders or restrictions due to COVID-19 (also known as 'lockdown')?", responses "yes" compared with "no".
Cumulative lockdown	Trichotomous variable based on total length of lockdown experienced by each state/territory. By 31 July 2021, the cumulative length of COVID-19 lockdown was greatest for the state of Victoria ("Vic", total 31 weeks); followed by the state of New South Wales ("NSW", total 15 weeks) and then all "other" states and territories (total range 10-12 weeks).
Mental health	
Caregiver mental health	6 items of the Kessler-6 (K6) assessing caregivers' self-reported anxiety and depressive symptoms encountered in the last 4 weeks. Scored on a 5-point Likert scale from 1 "none of the time" to 5 "all of the time". Summarized into (a) a continuous total score, and (b) a binary variable indicating "poor mental health" (total score 19 or more) compared with not (total score 6-18).[13]
Impact of the pandemic on mental health	A 5-point item adapted from UK Young Minds Matter Study,[14] describing the impact of COVID-19 on mental health, dichotomized into negative ("small negative/large negative") compared with positive ("none/small positive/large positive"). Reported by caregivers for (a) themselves and (b) each child.

Confidential: For Review Only

RESULTS

In June 2020, n=2020 of 2697 (74.9%) caregivers approached provided data for themselves and 3411 children. In September 2020, 1434/1769 (81.1%) caregivers provided data for 2553 children. In July 2021, 2508/3925 (63.9%) provided data for 4327 children. Table 2 presents the sample characteristics for each survey. There were some differences between surveys in characteristics such as the proportion of respondents caring for young children, caregiver gender, sole caregiving, and SEIFA; characteristics that were used to create the sample weights (see *Analysis*).

For the three surveys overall, children's mean age was 9.3 years (standard deviation (SD) 5.0 years) and 47.8% were female. Caregivers' mean age was 42.2 years (SD 9.1 years), 54.0% were female, and each caregiver cared for 1.7 children on average (SD 0.8). One-quarter were sole caregivers, 56.6% had a university degree, 25.6% were born outside Australia, and 22.4% spoke a language other than English at home. Twenty-eight percent of caregivers reported job/income loss due to COVID-19; 17.2% reported low household income and 29.3% were unable to afford at least one essential item in the month prior. The proportions of respondents in lockdown were 0% (June 2020), 29.2% (September 2020) and 56.5% (July 2021).

Table 3 presents the weighted mental health experiences for each survey, separately by characteristics. When considering patterns over time, the proportions of respondents who reported poor mental health according to the K6 was similar between surveys. Negative mental health impacts for caregivers became more common between June 2020 and July 2021, although there were no consistent patterns between June 2020 and September 2020, or between September 2020 and July 2021. In contrast, there was a clear pattern of increasingly common negative mental health impacts for children across the three waves of data collection.

Confidential: For Review Only

When considering differences between subgroups, negative mental health impacts for caregivers and children were more common when reported by female than male caregivers, and for older compared with younger children, but were similar between child genders. Poor mental health (K6) was more common for groups typically experiencing more social adversity, such as sole caregiving compared with multi-caregiver households, having a home language other than English (but not for country of birth), and several financial and lockdown characteristics (lower SEIFA and income, couldn't afford essentials, and cumulative or current lockdown). In contrast with the K6, negative mental health impacts for caregivers and children were more common for families experiencing greater social advantage, such as multi-caregiver (compared with sole caregiver) households and higher (compared with lower) education, SEIFA, and income. The proportions of respondents reporting all three negative mental health measures increased for families who had lost jobs or income due to the pandemic, and with cumulative lockdown.

Figure 1 shows that the three measures of negative mental health experiences increased for Victorian and NSW families by July 2021 compared with June 2020. Increases in the proportions of caregivers reporting poor mental health (K6) aligned with the second extended lockdowns; for Victorians this was September 2020 and for NSW it was July 2021 (supplementary data (a)). In contrast, there was weak evidence of change in the proportions of caregivers in Other states/territories reporting poor mental health (K6) over time. The proportions of caregivers and children experiencing negative mental health impacts due to the pandemic increased from June 2020 to July 2021 (supplementary data (b and c)). Increased proportions of Victorians reported negative impacts in September 2020 and again in July 2021. More caregivers from NSW and the Other states/territories self-reported negative impacts in July 2021 compared with June or September 2020.

Confidential: For Review Only

Table 2. Sample characteristics by survey, described with the number of respondents (proportion).

		Number of respondents (%)		
**Characteristic**	**Subgroup**	**June 2020**	**Sept 2020**	**July 2021**
Child		**N=3411**	**N=2553**	**N=4327**
Age in years	0-4	628 (18.4)	520 (20.4)	1291 (29.8)
	5-11	1356 (39.8)	1153 (45.2)	1705 (39.4)
	12-17	1427 (41.8)	880 (34.5)	1331 (30.8)
Gender	F	1664 (48.7)	1242 (48.7)	2013 (46.5)
	M	1747 (51.2)	1311 (51.4)	2314 (53.5)
Caregiver		**N=2020**	**N=1434**	**N=2508**
Gender	F	1003 (49.7)	758 (52.9)	1458 (58.1)
	M	1017 (50.4)	676 (47.1)	1050 (41.9)
Sole carer	Yes	462 (22.9)	282 (19.7)	704 (28.1)
	No	1558 (77.1)	1152 (80.3)	1804 (71.9)
Education [a]	Y12	326 (16.1)	239 (16.7)	422 (16.8)
	Cert.	518 (25.6)	390 (27.2)	693 (27.6)
	Uni.	1176 (58.2)	805 (56.1)	1393 (55.5)
Born outside Australia	Yes	546 (27.4)	329 (23.4)	622 (25.4)
	No	1444 (72.6)	1078 (76.6)	1827 (74.6)
Home language other than English	Yes	413 (20.5)	324 (22.6)	600 (23.9)
	No	1607 (79.6)	1110 (77.4)	1908 (76.1)
Socio-economic				
SEIFA quintile (1=most disadvantage, 5=least disadvantage)	1	213 (10.6)	172 (12.0)	327 (13.0)
	2	273 (13.5)	245 (17.1)	391 (15.6)
	3	370 (18.3)	250 (17.4)	511 (20.4)
	4	457 (22.7)	319 (22.3)	571 (22.8)
	5	705 (34.9)	448 (31.2)	707 (28.2)
Low income (<$1000) [c]	Yes	254 (14.7)	212 (16.9)	427 (19.2)
	No	1480 (85.3)	1040 (83.1)	1782 (80.7)
Couldn't afford essential items [d]	Yes	527 (26.1)	400 (27.9)	822 (32.8)
	No	1493 (73.9)	1034 (72.1)	1686 (67.2)
Pandemic experience				
Job/income loss [b]	Yes	559 (27.7)	381 (26.6)	711 (28.4)
	No	1461 (72.3)	1053 (73.4)	1797 (71.7)
Current lockdown [e]	Yes	0 (0)	419 (29.2)	1416 (56.5)
	No	2020 (100)	1015 (70.8)	1092 (43.5)
State as a proxy for cumulative lockdown (Vic=most, Other=least) [e]	Vic	612 (30.3)	473 (33.0)	746 (29.7)
	NSW	617 (30.5)	449 (31.3)	791 (31.5)
	Other	791 (39.2)	512 (35.7)	971 (38.7)

Vic: Victoria, NSW: New South Wales. Data were weighted using national demographic distributions for caregiver age, gender, family structure (sole-caregiving, number of children and any under 5 years), state/territory and Socio-Economic Indexes for Areas Index of Relative Disadvantage (SEIFA).

[a] Highest education coded as Year 12 or less (up to the end of high/secondary school; Y12); vocational training certificate ("cert."); or university degree ("uni").

[b] Job loss by one or two adults, or reduction in income, due to COVID-19.

[c] Low-income defined according to Australian definitions of income-poverty. Missing 767 caregivers who preferred not to report income.

[d] Any one or more of mortgage or rent; electricity, gas, water bills; food; healthcare; prescription medicines; home or car insurance; mobile phone bills; internet, in the last month.

[e] No Australians were in lockdown for the June 2020 Poll, so the number/proportion is for the whole cohort. For the September 2020 Poll, only residents of metropolitan Melbourne in the state of Victoria were in lockdown (Y) compared with all other Australians (N). In July 2021, many states/territories were going in and out of

Confidential: For Review Only

lockdown, and this question was asked directly (Y/N). Overall, Victorians experienced the most (cumulative) lockdown, followed by NSW, followed by Other states and territories.

Confidential: For Review Only

Table 3. Mental health experiences by sample characteristic and survey, described with the number of respondents (weighted proportion).

		Poor caregiver mental health (K6) [f]			Negative impact on caregiver [g]			Negative impact on child [g]		
**Characteristic**	**Subgroup**	**June 2020 N=2020**	**Sept 2020 N=1434**	**July 2021 N=2508**	**June 2020 N=2020**	**Sept 2020 N=1434**	**July 2021 N=2508**	**June 2020 N=3411**	**Sept 2020 N=2553**	**July 2021 N=4327**
Overall	All	264 (17.4)	247 (19.5)	541 (18.9)	949 (47.4)	708 (50.3)	1490 (60.0)	1055 (25.6)	884 (33.1)	1807 (44.0)
Child										
Age in years	0-4	-	-	-	-	-	-	81 (12.8)	101 (19.9)	330 (24.2)
	5-12	-	-	-	-	-	-	440 (32.3)	414 (37.3)	789 (46.9)
	13-17	-	-	-	-	-	-	534 (38.2)	369 (43.7)	688 (51.5)
Gender	F	-	-	-	-	-	-	525 (25.1)	438 (32.8)	876 (45.5)
	M	-	-	-	-	-	-	530 (26.0)	446 (33.4)	931 (42.7)
Caregiver										
Gender	F	153 (18.3)	110 (18.0)	338 (20.5)	489 (49.6)	410 (53.9)	920 (63.7)	512 (25.9)	521 (35.1)	1099 (45.7)
	M	111 (15.8)	137 (21.8)	203 (16.6)	460 (44.1)	298 (44.7)	570 (54.7)	543 (25.1)	363 (30.0)	708 (41.6)
Sole carer	Yes	137 (36.1)	100 (36.5)	268 (32.6)	199 (42.7)	99 (36.2)	335 (44.3)	190 (24.3)	144 (27.1)	397 (30.9)
	No	127 (10.2)	147 (14.8)	273 (15.0)	750 (49.2)	609 (54.1)	1155 (64.5)	865 (26.0)	740 (34.8)	1410 (47.9)
Education [a]	Y12	55 (21.7)	61 (26.9)	124 (15.8)	141 (40.2)	98 (42.9)	211 (47.3)	148 (21.2)	115 (24.3)	293 (39.2)
	Cert.	73 (21.4)	65 (19.9)	152 (20.0)	244 (48.7)	204 (56.1)	418 (64.6)	293 (28.6)	264 (34.9)	524 (51.1)
	Uni.	136 (15.8)	121 (16.9)	265 (17.5)	564 (48.8)	406 (49.6)	861 (61.5)	614 (25.3)	505 (34.9)	990 (41.8)
Born outside Australia	Yes	58 (12.5)	40 (11.7)	124 (16.9)	243 (42.4)	169 (49.2)	379 (60.0)	253 (23.0)	184 (32.0)	396 (40.2)
	No	198 (18.9)	198 (21.0)	405 (19.6)	694 (49.4)	530 (50.8)	1080 (60.1)	789 (26.8)	695 (33.7)	1375 (45.3)
Home language other than English	Yes	80 (20.9)	79 (22.5)	176 (25.1)	172 (40.6)	154 (47.6)	337 (58.9)	178 (21.7)	181 (31.7)	352 (39.5)
	No	184 (16.3)	168 (18.6)	365 (17.0)	777 (49.4)	554 (51.1)	1153 (58.9)	877 (26.8)	703 (33.5)	1455 (45.4)
Socio-economic										
SEIFA quintile (1=most disadvantage, 5=least disadvantage)	1	41 (22.1)	35 (20.7)	97 (26.1)	86 (41.4)	77 (47.4)	172 (56.0)	115 (27.1)	90 (31.3)	223 (42.0)
	2	50 (20.5)	53 (25.6)	99 (21.4)	125 (46.1)	117 (51.3)	215 (60.6)	133 (21.1)	157 (32.6)	262 (45.1)
	3	49 (16.2)	41 (19.3)	114 (17.9)	186 (53.1)	129 (51.9)	295 (58.7)	191 (27.3)	170 (34.5)	350 (40.8)
	4	66 (17.9)	54 (20.8)	108 (16.0)	205 (44.1)	149 (49.6)	350 (62.4)	242 (26.6)	172 (31.0)	392 (42.8)
	5	58 (9.4)	64 (11.1)	123 (16.5)	347 (51.7)	236 (51.0)	458 (67.1)	374 (26.3)	295 (35.7)	580 (52.9)
Low income (<$1000) [c]	Yes	69 (27.5)	58 (31.3)	171 (29.5)	120 (53.4)	89 (45.6)	230 (51.2)	111 (26.1)	133 (33.3)	271 (35.3)
	No	165 (15.5)	167 (17.3)	326 (16.6)	686 (45.2)	507 (49.2)	1064 (61.0)	774 (24.8)	631 (32.4)	1292 (44.6)

Confidential: For Review Only

		Poor caregiver mental health (K6) [f]			Negative impact on caregiver [g]			Negative impact on child [g]		
**Characteristic**	**Subgroup**	**June 2020 N=2020**	**Sept 2020 N=1434**	**July 2021 N=2508**	**June 2020 N=2020**	**Sept 2020 N=1434**	**July 2021 N=2508**	**June 2020 N=3411**	**Sept 2020 N=2553**	**July 2021 N=4327**
Couldn't afford essential items [d]	Yes	359 (37.4)	156 (38.4)	341 (35.5)	263 (50.3)	165 (44.8)	469 (59.2)	270 (26.5)	236 (33.9)	586 (42.6)
	No	96 (8.8)	91 (12.3)	200 (11.2)	686 (46.2)	543 (52.4)	1021 (60.4)	785 (25.1)	648 (32.8)	1221 (44.7)
Pandemic experience										
Job/income loss [b]	Yes	96 (23.7)	93 (27.5)	218 (28.8)	306 (51.0)	224 (59.5)	486 (71.1)	305 (25.0)	297 (40.7)	613 (55.8)
	No	168 (15.1)	288 (16.4)	323 (15.3)	643 (46.1)	484 (46.8)	1004 (55.9)	750 (25.7)	587 (30.2)	1194 (39.7)
Current lockdown [e]	Yes	n/a	83 (24.0)	353 (23.5)	n/a	240 (58.1)	867 (62.9)	n/a	355 (44.1)	1114 (49.6)
	No	264 (17.4)	164 (17.7)	188 (13.3)	949 (47.4)	468 (47.3)	623 (56.4)	1055 (25.6)	529 (28.7)	693 (37.3)
State as a proxy for cumulative lockdown (Vic=most, Other=least) [e]	Vic	85 (17.7)	93 (23.3)	200 (25.1)	284 (48.9)	266 (57.5)	483 (66.4)	321 (23.8)	389 (42.5)	626 (51.4)
	NSW	96 (21.6)	78 (18.6)	188 (22.0)	307 (49.2)	213 (49.0)	477 (60.8)	306 (25.9)	267 (34.4)	603 (48.9)
	Other	83 (12.4)	76 (16.6)	153 (11.2)	358 (43.9)	229 (44.1)	530 (54.4)	428 (26.9)	228 (21.0)	578 (33.7)

Vic: Victoria, NSW: New South Wales. proportions were weighted using national demographic distributions for caregiver age, gender, family structure (sole-caregiving, number of children and any under 5 years), state/territory and Socio-Economic Indexes for Areas Index of Relative Disadvantage (SEIFA). .

[a] Highest education coded as Year 12 or less (up to the end of high/secondary school; Y12); vocational training certificate ("cert."); or university degree ("uni").

[b] Job loss by one or two adults, or reduction in income, due to COVID-19.

[c] Low-income defined according to Australian definitions of income-poverty. Missing 767 caregivers who preferred not to report income.

[d] Any one or more of mortgage or rent; electricity, gas, water bills; food; healthcare; prescription medicines; home or car insurance; mobile phone bills; internet, in the last month.

[e] No Australians were in lockdown for the June 2020 Poll, so the proportion is for the whole cohort (see 'Overall' at top of Table). For the September 2020 Poll, only residents of metropolitan Melbourne in the state of Victoria were in lockdown (Y) compared with all other Australians (N). In July 2021, many states/territories were going in and out of lockdown, and this question was asked directly (Y/N). Overall, Victorians experienced the most (cumulative) lockdown, followed by NSW, followed by Other states and territories.

[f] Kessler-6 (K6) dichotomized into a binary "poor mental health" (total score 19 or more) compared with not (total score 6-18).

[g] Dichotomised into negative ("small negative/large negative") compared with positive ("none/small positive/large positive").

Confidential: For Review Only

DISCUSSION

In the only nationally representative and repeated cross-sectional survey of Australian families over 12 months of the COVID-19 pandemic, there were unequal distributions of mental health impacts based on caregiver gender, child age, socioeconomic and pandemic-related characteristics. As hypothesised, negative mental health impacts were more common for female compared with male caregivers, and older children compared with younger children. However, poor caregiver mental health (K6) was similar between female and male genders. Job or income loss due to the pandemic was detrimental for caregiver and child mental health. While poor caregiver mental health (K6) was more common for families experiencing greater social adversity, reported negative impacts were more common for more socially advantaged groups. Caregivers in states/territories who experienced the least cumulative lockdown reported similar K6 scores over time. Otherwise, poor mental health and negative mental health impacts increased over the course of the three surveys and with cumulative lockdown.

Comparable data on the mental health experiences specific to caregivers of children during the pandemic are limited. Aknin and colleagues' review of mental health experiences during COVID-19 suggest that levels of psychological distress in general adult populations may have declined from an early peak in the first months of the pandemic.[4] In our study, a similar improvement was found for the mental health (K6) of Australian caregivers in the states/territories that experienced the least lockdown. However, our data demonstrated evidence that negative mental health experiences increased over time and were strongly related to cumulative lockdown. These findings align with and extend data from the Pulse of the Nation (TPTN), a weekly, cross-sectional survey of representative Australian adult samples throughout the pandemic.[15] Like our study, TPTN data indicated that mental distress in adults (measured with a single item that highly correlated with the K6) increased during

https://mc.manuscriptcentral.com/bmjpo

2020, which the authors describe as driven by increasing financial stress.[15] Notably, increases in mental distress were highest for parents, tripling from 8% pre-pandemic to 24% during the pandemic.[15] While the RCH Poll lacked pre-pandemic data, substantially more caregivers reported poor mental health on the K6 (average 18%) in the Polls throughout the pandemic than representative Australian adult data collected pre-pandemic (8% in 2017) or during the first national lockdown (11%).[16] Our findings of increasing negative mental health impacts for children are consistent with the emerging reviews of the published evidence.[9]

Strengths of our study included the large cross-sectional and nationally representative surveys, which employed strong methodology (surveys piloted and included the validated K6); collected data on caregiver and child mental health; surveyed female and male caregivers; and achieved good response rates. In other Polls, indicators (frequency/prevalence) across a range of topics are almost universally consistent with more traditionally obtained estimates, providing support for the sample selection and survey administration methods. By July 2021, we found that negative mental health impacts were reported for half of children and two-thirds of adults. This highlights how general pandemic stress, and disruption to family life including employment, routines, and social interactions, can adversely affect caregivers and children. Despite Australia's low incidence of COVID-19, the increases in psychological distress are consistent with data from countries with high infection rates and the increases in distress are sizable.[4 6 8] This pattern has been described for previous health crises; following the H1N1 pandemic, a quarter of parents and 30% of children who were required to isolate met criteria for PTSD symptoms.[17]

This study also had limitations. The reliance on caregiver-report, from only one caregiver per household, means the child rating may be biased by caregiver perception. We did not collect a validated measure of children's mental health that would provide a measure of clinical

Confidential: For Review Only

impact. The finding of different social gradients for the K6 compared with the negative impact items may represent differing expectations and emphasises the need for validated measures for anticipating the longer-term consequences of lockdown, which ideally would be collected from all caregivers and children directly. The RCH Poll only samples adults who are 18 years and older, so younger caregivers are missing, and a significant proportion of caregivers did not report family income. However, the weighted and adjusted analyses support the generalisability of findings to the broader population of Australian families raising children and young people.

Australia's reliance on lockdown has meant that caregivers had to concurrently parent, work, and facilitate remote schooling. Our findings show that, despite the low disease incidence, the pandemic experience has undermined caregivers' and children's mental health. Understanding specific risk factors for poorer mental health over time was beyond the scope of the cross-sectional data. However, our findings support the inequities evident in existing research. As researchers have suggested,[4 6 8] understanding and prioritising population risk groups should be the subject of ongoing research. Clearly, pandemic response and recovery planning must prioritise and incorporate mental health supports. To this end, we endorse the recommendations proposed by Aknin and colleagues.[6] These include prioritizing safe access to childcare and schools; greater investment in evidence-informed mental health services including telehealth, and its integration with social care systems; and mental health promotion across communities such as work and schools. They also emphasise the importance of monitoring of mental health. As our findings demonstrate, mental health is changing during the pandemic. Understanding families' experiences is necessary to inform policy effort with a greater level of precision, so that we can respond to the evolving mental health needs of children and their families.

Confidential: For Review Only

FIGURE LEGEND

Figure 1. Estimated probabilities over time (by survey) and by state/territory (as a proxy for cumulative lockdown) adjusted for potential confounders for (a) caregiver K6, and negative mental health impacts for the (b) caregiver and (c) children.

ACKNOWLEDGMENTS AND FUNDING

We thank all families who took part in the Royal Children's Hospital National Child Health Polls. The National Child Health Polls are funded by The Royal Children's Hospital Foundation (Grant #n/a). This work was supported by The Healthier Wealthier Families project, which was supported by the Helen Macpherson Smith Trust Impact Grant #9523 and The Corella Fund (Grant #n/a). The Murdoch Children's Research Institute (MCRI) administered research grants for the work and provided infrastructural support (as study sponsor) to its staff but played no role in the conduct or analysis of the research. Research at the MCRI is supported by the Victorian Government's Operational Infrastructure Support Program. Author AP was supported by The Erdi Foundation Child Health Equity (COVID-19) Scholarship (Grant# n/a). Author SG was supported by a NHMRC Practitioner Fellowship (#1155290).

COMPETING INTERESTS

None to declare.

AUTHOR CONTRIBUTIONS

Anna Price: Conceptualization, Validation, Formal analysis, Investigation, Resources, Data curation, Writing – Original Draft, Writing - Review & Editing, Visualization, Supervision, Project administration, Funding acquisition.

Mary-Anne Measey: Conceptualization, Methodology, Validation, Investigation, Data curation, Writing - Review & Editing.

Monsurul Hoq: Methodology, Validation, Formal analysis, Investigation, Data curation, Writing - Review & Editing.

Anthea Rhodes: Conceptualization, Methodology, Investigation, Resources, Writing - Review & Editing, Supervision, Project administration, Funding acquisition.

Sharon Goldfeld: Conceptualization, Methodology, Investigation, Resources, Writing - Review & Editing, Supervision, Funding acquisition.

AR and SG share senior authorship.

DATA SHARING STATEMENT

The data underlying the results presented in the study are available from the RCH Child Health Poll, please contact child.healthpoll@rch.org.au.

Confidential: For Review Only

REFERENCES

1. Hale T, Angrist, Noam. , Goldszmidt R, Kira B, Petherick A, Phillips T, et al. A global panel database of pandemic policies (Oxford COVID-19 Government Response Tracker). Nature Human Behaviour. https://doi.org/10.1038/s41562-021-01079-8. Retrieved from: https://ourworldindata.org/grapher/covid-stringency-index [Online Resource] (2021). 2021.
2. Australian Department of Health. Coronavirus (COVID-19) at a glance - 31 July 2021. https://www.health.gov.au/sites/default/files/documents/2021/08/coronavirus-covid-19-at-a-glance-31-july-2021_0.pdf.
3. Mathieu E, Ritchie H, Ortiz-Ospina E, et al. A global database of COVID-19 vaccinations. *Nature human behaviour* 2021:1-7.
4. Aknin L, De Neve J-E, Dunn E, et al. Mental health during the first year of the COVID-19 pandemic: A review and recommendations for moving forward. *Perspectives on Psychological Science* 2021
5. Centre for Mental Health. Addressing inequities in mental health research exacerbated by Covid-19. A report by the Mental Health Research Group. 6 July 2021 [https://www.centreformentalhealth.org.uk/publications/fit-for-purpose, last accessed 24 Nov 2021].
6. Samji H, Wu J, Ladak A, et al. Review: Mental health impacts of the COVID-19 pandemic on children and youth – a systematic review. *Child and Adolescent Mental Health*;n/a(n/a) doi: https://doi.org/10.1111/camh.12501
7. Jones EAK, Mitra AK, Bhuiyan AR. Impact of COVID-19 on Mental Health in Adolescents: A Systematic Review. *Int J Environ Res Public Health* 2021;18(5) doi: 10.3390/ijerph18052470 [published Online First: 2021/04/04]
8. Racine N, McArthur BA, Cooke JE, et al. Global Prevalence of Depressive and Anxiety Symptoms in Children and Adolescents During COVID-19: A Meta-analysis. *JAMA Pediatrics* 2021;175(11):1142-50. doi: 10.1001/jamapediatrics.2021.2482
9. Meherali S, Punjani N, Louie-Poon S, et al. Mental Health of Children and Adolescents Amidst COVID-19 and Past Pandemics: A Rapid Systematic Review. *International Journal of Environmental Research and Public Health* 2021;18(7):3432.
10. Nikolaidis A, Paksarian D, Alexander L, et al. The Coronavirus Health and Impact Survey (CRISIS) reveals reproducible correlates of pandemic-related mood states across the Atlantic. *Sci Rep* 2021; 11(1). (accessed 2021/04//).
11. Davidson P, Saunders P, Bradbury B, et al. Poverty in Australia 2020: Part 1, Overview. ACOSS/UNSW Poverty and Inequality Partnership Report No. 3, Sydney: ACOSS.; 2020.
12. Wilkins R, Lass I. The Household, Income and Labour Dynamics in Australia Survey: Selected Findings from Waves 1 to 16. Melbourne Institute: Applied Economic & Social Research, University of Melbourne., 2018.
13. Furukawa TA, Kessler RC, Slade T, et al. The performance of the K6 and K10 screening scales for psychological distress in the Australian National Survey of Mental Health and Well-Being. *Psychol Med* 2003;33(2):357-62. doi: 10.1017/s0033291702006700 [published Online First: 2003/03/08]
14. Young Minds. Coronavirus: Impact on young people with mental health needs. https://youngminds.org.uk/media/3708/coronavirus-report_march2020.pdf. 2020.
15. Broadway B, Payne A, A, Salamanca N. Coping with COVID-19: Rethinking Australia. Melbourne Institute: Applied Economic & Social Research, the University of Melbourne., 2020.
16. Edwards B, Biddle N, Gray M, et al. Initial impacts of COVID-19 on mental health in Australia. 2020
17. Sprang G, Silman M. Posttraumatic stress disorder in parents and youth after health-related disasters. *Disaster medicine and public health preparedness* 2013;7(1):105-10.

Confidential: For Review Only

Supplementary data: estimated probabilities over time (by survey) and by state/territory (as a proxy for cumulative lockdown) for the three mental health measures (graphed in Figure 1)

(a) Poor caregiver mental health (Kessler-6)

```
Generalized linear models                         Number of obs   =      5,854
Optimization     : MQL Fisher scoring             Residual df     =      5,840
                   (IRLS EIM)                     Scale parameter =          1
Deviance         =  9280572.574                   (1/df) Deviance =   1589.139
Pearson          =  10441040.84                   (1/df) Pearson  =   1787.849

Variance function: V(u) = u*(1-u)                 [Bernoulli]
Link function    : g(u) = u                       [Identity]

                                                  BIC             =    9229911
```

parentk6tot_d	Risk diff.	Semirobust std. err.	z	P>|z|	[95% conf.	interval]
state_r						
NSW	.0718525	.0258502	2.78	0.005	.021187	.1225179
Vic	.0494101	.023712	2.08	0.037	.0029355	.0958847
poll						
W2 sept 2020	.0450288	.0269215	1.67	0.094	-.0077365	.097794
W3 july 2021	.0080103	.0161058	0.50	0.619	-.0235565	.0395772
state_r#poll						
NSW#W2 sept 2020	-.0623205	.0408931	-1.52	0.128	-.1424695	.0178284
NSW#W3 july 2021	.0292478	.0336068	0.87	0.384	-.0366202	.0951158
Vic#W2 sept 2020	.0310635	.043261	0.72	0.473	-.0537265	.1158535
Vic#W3 july 2021	.0784145	.0331736	2.36	0.018	.0133954	.1434336
sex	.0010905	.0125578	0.09	0.931	-.0235223	.0257033
q2a	.2219676	.0232243	9.56	0.000	.1764489	.2674863
education_c3	.0207678	.0094677	2.19	0.028	.0022114	.0393241
Score	-.0002257	.0000974	-2.32	0.020	-.0004166	-.0000348
cald	.0236198	.0130602	1.81	0.071	-.0019777	.0492173
_cons	.2722676	.1000927	2.72	0.007	.0760895	.4684457

(b) Negative impacts of the pandemic on caregiver mental health

```
Generalized linear models                         Number of obs   =      5,854
Optimization     : MQL Fisher scoring             Residual df     =      5,840
                   (IRLS EIM)                     Scale parameter =          1
Deviance         =  13913646.93                   (1/df) Deviance =   2382.474
Pearson          =  10441040.98                   (1/df) Pearson  =   1787.849

Variance function: V(u) = u*(1-u)                 [Bernoulli]
Link function    : g(u) = u                       [Identity]

                                                  BIC             =   1.39e+07
```

w16q52_d	Risk diff.	Semirobust std. err.	z	P>|z|	[95% conf.	interval]
state_r						
NSW	.0600002	.0401823	1.49	0.135	-.0187556	.1387561
Vic	.0568247	.0407174	1.40	0.163	-.0229799	.1366293
poll						
W2 sept 2020	-.0051744	.043287	-0.12	0.905	-.0900155	.0796666
W3 july 2021	.1272643	.0357338	3.56	0.000	.0572273	.1973012
state_r#poll						
NSW#W2 sept 2020	-.0053991	.0603809	-0.09	0.929	-.1237435	.1129454
NSW#W3 july 2021	-.0234892	.0514006	-0.46	0.648	-.1242325	.0772541
Vic#W2 sept 2020	.0756745	.0606002	1.25	0.212	-.0430996	.1944487
Vic#W3 july 2021	.0442841	.0514682	0.86	0.390	-.0565917	.1451599
sex	-.1035235	.0187399	-5.52	0.000	-.1402531	-.066794
q2a	-.1378773	.0240192	-5.74	0.000	-.184954	-.0908005
education_c3	-.0199496	.0130221	-1.53	0.126	-.0454725	.0055733
Score	.0002641	.0001445	1.83	0.068	-.000019	.0005472
cald	-.059999	.0193952	-3.09	0.002	-.0980129	-.0219852
_cons	.2869052	.15113	1.90	0.058	-.0093041	.5831144

Confidential: For Review Only

```
--------------------------------------------------------------------------------
```

(c) Negative impacts of the pandemic on child mental health

```
Generalized linear models                         Number of obs     =     10,109
Optimization     : MQL Fisher scoring             Residual df       =     10,093
                   (IRLS EIM)                     Scale parameter =            1
Deviance         =  13214524.01                   (1/df) Deviance =   1309.276
Pearson          =  10717625.13                   (1/df) Pearson  =   1061.887

Variance function: V(u) = u*(1-u/1)               [Binomial]
Link function    : g(u) = u                       [Identity]

                                                  BIC             =   1.31e+07

                              (Std. err. adjusted for 4,877 clusters in responseid)
--------------------------------------------------------------------------------
                 |             Semirobust
        w16q54_d | Risk diff.   std. err.      z    P>|z|     [95% conf. interval]
-----------------+--------------------------------------------------------------
         state_r |
             NSW |  -.0091262   .0346798    -0.26   0.792    -.0770974     .058845
             Vic |  -.0249992   .0323287    -0.77   0.439    -.0883623    .0383639
                 |
            poll |
    W2 sept 2020 |  -.0644975   .0345009    -1.87   0.062     -.132118    .0031231
    W3 july 2021 |   .0793999   .0316666     2.51   0.012     .0173345    .1414652
                 |
    state_r#poll |
NSW#W2 sept 2020 |   .1487946   .0523181     2.84   0.004     .0462531    .2513362
NSW#W3 july 2021 |   .1413132    .046868     3.02   0.003     .0494535    .2331728
Vic#W2 sept 2020 |   .2357508   .0501326     4.70   0.000     .1374927    .3340089
Vic#W3 july 2021 |   .1847532   .0441666     4.18   0.000     .0981883    .2713181
                 |
            q1c_ |  -.0048253   .0134087    -0.36   0.719    -.0311058    .0214552
             sex |  -.0455852   .0167225    -2.73   0.006    -.0783606   -.0128098
    parentk6tot_d |   .0411459   .0249218     1.65   0.099      -.0077    .0899918
             q2a |  -.0709401   .0208751    -3.40   0.001    -.1118546   -.0300255
    education_c3 |  -.0062353    .011399    -0.55   0.584     -.028577    .0161063
           Score |    .000286   .0001372     2.08   0.037     .0000171    .0005549
            cald |  -.0375271   .0178451    -2.10   0.035    -.0725028   -.0025514
           _cons |   .0359978   .1433662     0.25   0.802    -.2449948    .3169904
--------------------------------------------------------------------------------
```

Confidential: For Review Only

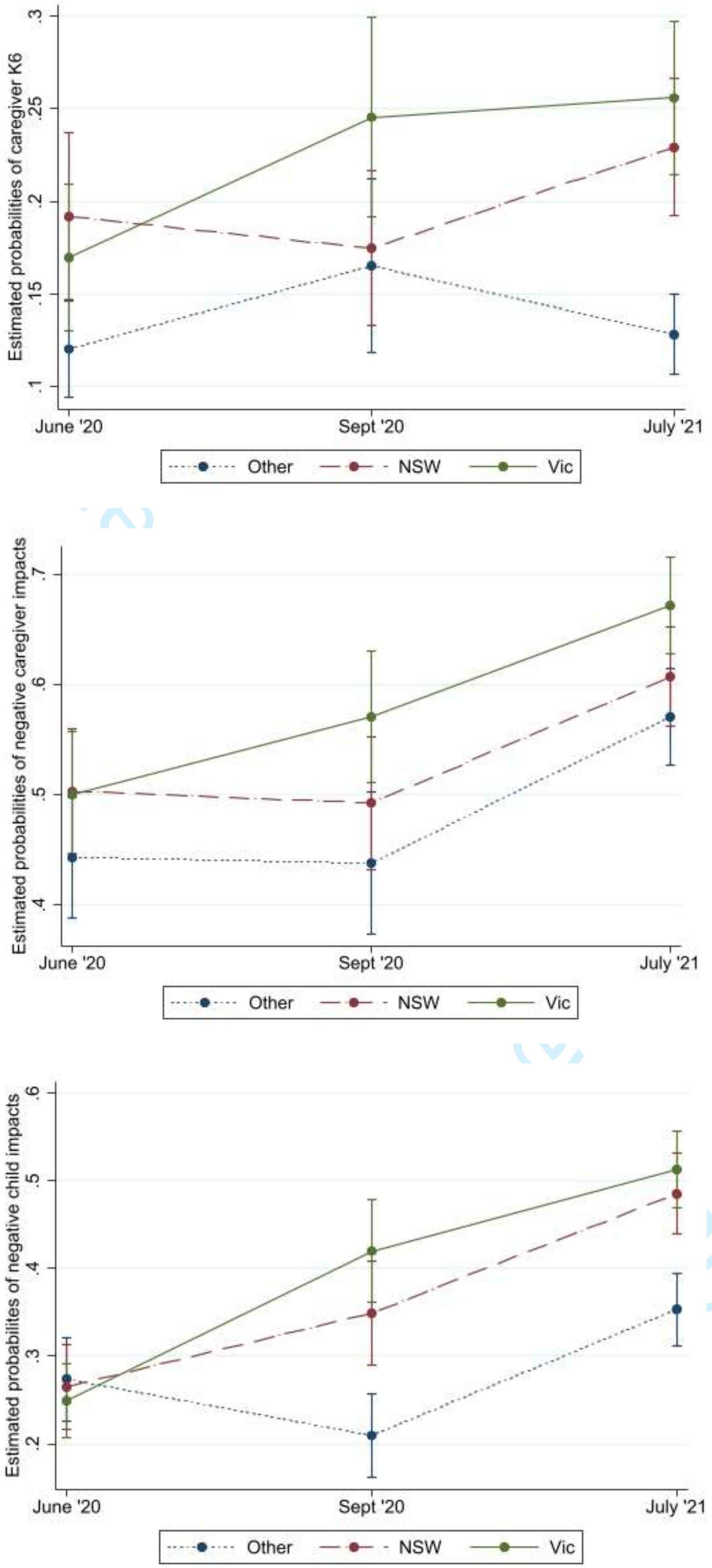
Estimated probabilities of caregiver K6
.1
.15
.2
.25
.3
June '20
Sept '20
July '21
Other
NSW
Vic
Estimated probabilities of negative caregiver impacts
.4
.5
.6
.7
June '20
Sept '20
July '21
Other
NSW
Vic
Estimated probabilites of negative child impacts
.2
.3
.4
.5
.6
June '20
Sept '20
July '21
Other
NSW
Vic

Response to reviewers (Previous MS ID: archdischild-2021-323101)

Reviewer Comment	Response
Editor	
The comments of the reviewer(s) are included at the bottom of this letter. Although the reviewers had no major criticisms, the editorial board could not justify continuing to publication given the volume and nature of other papers being considered	Thank you for the Reviewers' thorough reviews and the invitation to transfer the manuscript to BMJ Paediatrics Open. To revise the paper from a short report to a full paper and respond to the Reviewer comments, the paper is substantially rewritten. As such, below we provide a summary of the key changes rather than the complete sections, which are tracked in the submission.
Reviewer #1	
1. This short report paper describes caregiver and child mental health (as reported by caregivers) during the COVID-19 pandemic in Australia across three time points (June 2020, September 2020 and July 2021). The strengths of this paper are that it uses data from a large, nationally representative sample, the results are well-represented and it is well-written and clear.	Thank you.
2. While the short report format might not lend itself to more complex statistical analyses (e.g., multiple regression), I do question the descriptive approach when a more sophisticated statistical approach might warrant greater insight in to hypothesised outcomes. Further, my recommendation is to focus in on the Australian experience of COVID-19, given Australia's experience of lockdown and COVID has been relatively mild during the study period	As suggested by the reviewer (and now that this short report is written into a longer format), we have added more sophisticated analysis to investigate the change in mental health measures over time. This included binomial regression models that investigated whether cumulative lockdown worsened the mental health measures over time, after adjusting for key covariates. Please see the Analysis section and Figures for full details.
3. Title<br>Suggestion to include "in Australia" in the title as this is an Australian specific study and may not reflect caregiver or child mental health outcomes globally	Revised as suggested.

Confidential: For Review Only

4. Introduction<br>Strong rationale for why we should be focusing on mental health. The authors review evidence for global mental health evidence (Aknin et al., 2021), yet could the authors provide a brief rationale for why it is important to look at Australia's experience, and what they might expect versus globally, given that Australia's lockdown (and COVID) experience has been relatively mild during the time period reviewed in the study.	As requested, we have added rationale to first and third paragraphs of the revised Introduction to describe how Australia's experience gives insight into mental health impacts of the pandemic mostly independent of the compounding impacts of the disease.
5. Could the authors specify in their hypotheses the direction of effects for demographic factors (e.g., caregiver gender (female), child age (younger). I assume they are predicting what is reported in the Aknin study – but it would be helpful to have this specified.	As requested, we have specified the hypotheses as follows: "Based on the gender, age and socioeconomic disparities identified in the published evidence reviews, we hypothesised that poor mental health and negative impacts of the pandemic on mental health would be more common: (i) for caregivers who identified as female compared with male, and older compared with younger children, (ii) for families experiencing greater socioeconomic adversity compared with more socioeconomic advantage and (iii) with cumulative (increasing total length) of lockdown"
6. Method<br>I am not aware of the UK Young Minds Matter study nor the measure which the authors report on (the impact of COVID-19 on caregiver's mental health health). Given this provides two of the main outcome measures of mental health I would recommend that the authors provide a reference for this (and take out another to meet the 5 references limit).	As requested, we have added the reference to the new Measures Table (Table 1):<br>Young Minds. Coronavirus: Impact on young people with mental health needs. https://youngminds.org.uk/media/3708/coronavirus-report_march2020.pdf. 2020
7. Analysis<br>Could the weighting be explained a little more briefly (e.g., in text under Table 1 if don't have word count) and also how the child data were "adjusted for family cluster".	As requested, we have revised and added more detail to the analysis section, as follows:<br>"To reduce effects of non-response and non-coverage and therefore approximate the population distributions of mental health experiences, the mental health measures were weighted

Confidential: For Review Only

	using ABS distributions of caregiver age, gender, family structure (sole-caregiving, number of children and any under 5 years), state/territory and SEIFA. Mental health measures were described with weighted proportions for each survey, overall and by demographic, socioeconomic and pandemic-related financial and lockdown characteristics. For each of the three mental health measures, the association between mental health measure and time of the survey, according to state/territory grouping (as a proxy for cumulative lockdown, see Table 1), were calculated using generalised linear models. The binomial models investigated whether cumulative lockdown worsened the mental health measures over time, after adjusting for caregiver gender, sole caregiver status, education, SEIFA, and cultural or linguistic diversity (measures described in Table 1). The child mental health model was additionally adjusted for poor caregiver mental health (Kessler-6, see Table 1) and clustering at the level of family. The estimated models were then used to predict probabilities of each mental health outcome, by state/territory grouping, at the three survey timepoints, adjusted for the covariates in the model."
8. Results<br>The authors report that demographic characteristics were "similar across the three surveys" yet based on calculations from the tables this is not always the case (e.g., sole caregivers as a proportion of overall sample varies between 18-28%. Given the proportion range is reported for other variables (e.g., job/loss income and low income households), I would suggest presenting a range for the other variables as it is misleading as presented in the text.	As the Reviewer notes, there were some differences in the sample characteristics between surveys. These were sole caregiving and proportion of young children. As such, we have revised the weighting to incorporate the population distributions of these two variables. We have revised the Results to present unweighted demographic characteristics (Table 2) followed by weighted mental health characteristics (Table 3).
9. How did the authors determine participants' lockdown status for the Sept 2020 poll? Did they ask participants to provide a postcode and then determine whether the postcode was in metro Melbourne? Please	As requested, definitions of lockdown status are added to the new Measures Table (Table 1). While regional Victorians were still experiencing a partial lockdown during the September poll, they

Confidential: For Review Only

specify. Also, based on my understanding, regional VIC was also in lockdown during the September period, not only metro Melbourne so some data adjustment may be needed.	were in Step 3 from 14 Sept (https://www.abc.net.au/news/2020-09-06/when-will-regional-victoria-coronavirus-lockdown-end-roadmaps/12634548) which was much reduced and so, to provide a conservative estimate of the experience of lockdown, they are classified as not in lockdown in the September 2020 Poll.
10. Discussion<br>The authors report that their findings align with the Pulse of the Nation survey – and that "notably, this increase in psychological distress is not reflected in general population data". These two sentences assume knowledge and need better explanation.	As requested, this has been clarified with the following addition:<br><br>"These findings align with and extend data from the Pulse of the Nation (TPTN), a weekly, cross-sectional survey of representative Australian adult samples throughout the pandemic. Like our study, TPTN data indicated that mental distress in adults (measured with a single item that correlated strongly with the K6) increased during 2020, which the authors describe as driven by increasing financial stress. Notably, increases in mental distress were highest for parents, tripling from 8% pre-pandemic to 24% during the pandemic."
11. Might the authors briefly compare the outcomes for Australia to data globally? How does Australia's experience compare and what does that mean for lockdown policies?	As requested, we have discussed the results in the context of the broader global literature, noting that comparable data are limited.
12. I do think the authors need to couch their findings within the descriptive approach taken – i.e., it is unknown whether mental health deteriorated in a statistical sense of the word and whether certain demographic characteristics were significantly associated with worse mental health. Indeed, the "unequal distribution of outcomes" might reflect pre-existing inequities pre-pandemic (e.g., low socioeconomic status associated with poorer mental health) and so effects of the pandemic are unknown.	Our reweighting and new analyses estimating proportions over time have refined this analysis, and now present statistical tests to support this statement.
Reviewer #2	

Confidential: For Review Only

13. This is a really important paper, clearly written, and will be of great interest to ADC readers. I have only a few minor suggestions.	Thank you
14. Take home messages in Abstract /What this study adds / Discussion: I appreciate that words are limited, but I thought that the description of the findings ("unequal distribution of mental health impacts based on caregiver gender, child age and family socioeconomic characteristics") undersold the findings. The results indicate that population-level mental health (as measured using a validated tool) and declines in mental health were worse for lower socio-economic groups, while perceived impacts of lockdown on caregiver and child mental health appeared to be greater among advantaged groups. This possibly speaks to the idea of differing expectations and emphasises the need for validated measures for anticipating the longer term consequences of lockdown?	We have incorporated the Reviewer's interpretation into the Abstract and the limitations section of the Discussion; however, we have retained the 'What this study adds' summary to meet the 25-word limit.
15. In the intro it would be helpful to briefly describe the impacts of lockdown on parents & children services - e.g. what happened to health visiting, early years learning, schooling etc. For those not familiar with the Australian context. And to include rates of covid in the general population in the last sentence of the first para.	As requested, the rates of COVID in Australia and a description of the impacts on services are added to first paragraph of the Introduction. We have also updated the Introduction and Discussion with recently published systematic reviews.
16. Methods: The initial description of the survey implies that data were possibly collected pre-lockdown (i.e. that this is an ongoing survey). This makes the reader wonder whether it would have been possible to look at how trends changed before/after lockdown, so may be worth clarifying.	As suggested, we have revised the Methods to include the following:<br>"Questions about mental health – the focus of the current study – were introduced after COVID-19 began and were collected in three surveys."
17. It is stated in the results that the sample characteristics were comparable across the three surveys (and therefore a description of the samples are presented, in the text, in a collapsed form). However it would be helpful for the reader to be able to judge for themselves how	As the Reviewer notes, there were some differences in the sample characteristics between surveys that have occurred by chance, but may have introduced confounding bias. These were sole caregiving and proportion of young children. As such, we have

Confidential: For Review Only

comparable the three surveys were (especially since collider bias has been a common criticism of surveys during covid) therefore please could weighted %s be included in table along with the Ns?	revised the weighting to incorporate the population distributions of these two variables. We have also revised our analysis and Results to describe the sample characteristics (N (unweighted %) in text and Table 2), followed by the weighted mental health experiences (N (weighted %) in text and Table 3).
18. The results don't really focus much on how population-level mental health prevalences *changed* in different groups across the period, despite the emphasis on this in the initial hypotheses. It would be interesting is this could be brought out a little more (and perhaps could replace the text description of the samples if more info is provided in Table 1?).	As described in the preceding comments, we have revised the weighting to better represent the population distribution and extended the analysis to include a regression of the mental health experiences over time. A description of the overall mental health experiences, referring to these analyses, is now integrated throughout.
19. Table: a description of the lockdown situation in NSW at the foot of the table would be helpful, since this is used as one of the comparison groups.	As requested, this is added in the new Measures Table (Table 1) and to the bottom of the Results Tables (Tables 2 and 3).

Confidential: For Review Only

BMJ Paediatrics Open

Child and caregiver mental health during 12 months of the COVID-19 pandemic in Australia: findings from national repeated cross-sectional surveys

Journal:	*BMJ Paediatrics Open*
Manuscript ID	bmjpo-2021-001390.R1
Article Type:	Original research
Date Submitted by the Author:	04-Apr-2022
Complete List of Authors:	Price, Anna; Murdoch Childrens Research Institute, Centre for Community Child Health; The University of Melbourne, Paediatrics<br>Measey, Mary-Anne; The Royal Children's Hospital Melbourne, General Medicine<br>Hoq, Monsurul; Murdoch Childrens Research Institute, Clinical Epidemiology and Biostatistics Unit<br>Rhodes, Anthea; Royal Children's Hospital Melbourne, General Medicine<br>Goldfeld, Sharon; Royal Children's Hospital Melbourne, Centre for Community Child Health
Keywords:	COVID-19, Psychology

SCHOLARONE™
Manuscripts

I, the Submitting Author has the right to grant and does grant on behalf of all authors of the Work (as defined in the below author licence), an exclusive licence and/or a non-exclusive licence for contributions from authors who are: i) UK Crown employees; ii) where BMJ has agreed a CC-BY licence shall apply, and/or iii) in accordance with the terms applicable for US Federal Government officers or employees acting as part of their official duties; on a worldwide, perpetual, irrevocable, royalty-free basis to BMJ Publishing Group Ltd ("BMJ") its licensees and where the relevant Journal is co-owned by BMJ to the co-owners of the Journal, to publish the Work in this journal and any other BMJ products and to exploit all rights, as set out in our licence.

The Submitting Author accepts and understands that any supply made under these terms is made by BMJ to the Submitting Author unless you are acting as an employee on behalf of your employer or a postgraduate student of an affiliated institution which is paying any applicable article publishing charge ("APC") for Open Access articles. Where the Submitting Author wishes to make the Work available on an Open Access basis (and intends to pay the relevant APC), the terms of reuse of such Open Access shall be governed by a Creative Commons licence – details of these licences and which Creative Commons licence will apply to this Work are set out in our licence referred to above.

Other than as permitted in any relevant BMJ Author's Self Archiving Policies, I confirm this Work has not been accepted for publication elsewhere, is not being considered for publication elsewhere and does not duplicate material already published. I confirm all authors consent to publication of this Work and authorise the granting of this licence.

Confidential: For Review Only

Child and caregiver mental health during 12 months of the COVID-19 pandemic in Australia: findings from national repeated cross-sectional surveys

Authors:
*Anna M. H. Price[1,2,3], Mary-Anne Measey[2, 4], Monsurul Hoq[5], Anthea Rhodes[2,3,6, ¶], Sharon Goldfeld[1,2,3,¶]

Affiliations^:
1. Centre for Community Child Health, The Royal Children's Hospital, Parkville, Vic
2. Population Health, Murdoch Children's Research Institute, Parkville, Vic
3. Department of Paediatrics, The University of Melbourne, Parkville, Vic
4. Health Services Research Unit, The Royal Children's Hospital, Parkville, Vic
5. Clinical Epidemiology and Biostatistics Unit, Murdoch children's Research Institute, Parkville, Vic
6. Department of General Medicine, The Royal Children's Hospital, Parkville, Vic

^All Australia,
*Corresponding author, anna.price@mcri.edu.au
¶ AR and SG are joint senior authors.

Word Count: 2826 words

Confidential: For Review Only

ABSTRACT

Background: There are calls for research into the mental health consequences of living through the COVID-19 pandemic. Australia's initial, effective suppression of COVID-19 offers insights into these indirect impacts in the relative absence of the disease. We aimed to describe the mental health experiences of Australian caregivers and children over 12 months, reporting differences related to demographic, socioeconomic and lockdown characteristics.

Methods: Data were from Australia's only nationally representative, repeated cross-sectional survey of caregivers with children (0-17 years). N=2020 caregivers participated in June 2020, N=1434 in September 2020, and N=2508 in July 2021. Caregivers reported their mental health (poor versus not, Kessler-6), and perceived impacts of the pandemic on theirs and their children's mental health (negative versus none/positive). Data were weighted to approximate population distributions of caregiver age, gender, sole-caregiving, number and ages of children, state/territory and neighbourhood-level disadvantage.

Results: Perceived impacts on mental health were more frequently negative for female (versus male) caregivers and older (versus younger) children. Poor caregiver mental health (K6) was more common for families experiencing socioeconomic adversity (especially financial), while perceived impacts were more frequently negative for more socially advantaged groups. Caregivers who experienced the least total lockdown reported similar K6 over time. Otherwise, poor mental health and perceived negative impacts increased over time with increasing total length of lockdown.

Conclusion: Despite Australia's low infection rates, the negative mental health experiences of the COVID-19 pandemic are real and concerning. Addressing poor mental health must be central to ongoing pandemic recovery efforts for families and children.

What is known about this topic?

- The global evidence shows that, for general adult populations, psychological distress peaked in the first months of the COVID-19 pandemic before appearing to improve.
- Less is known about mental health over time of living through the pandemic, especially for caregivers and children. There are urgent calls for research.
- Due to low infection rates, Australia's experience can provide insight into the mental health impacts of lockdown with minimal compounding harms of the virus.

What this study adds:

- From June 2020 to July 2021, Australia's lockdowns were detrimental for caregiver and child mental health.
- Negative mental health experiences differed by caregiver gender, child age and family socioeconomic characteristics.
- Pandemic response and recovery planning must consider both family mental health and socioeconomic security.

Confidential: For Review Only

INTRODUCTION

During the first eighteen months of the COVID-19 pandemic, balancing the direct harms of the virus with the indirect harms of public health restrictions was a global challenge. Australia enacted some of the strictest public health measures in the world to effectively suppress infection.[1] By 31 July 2021, the measures successfully contained infection to an overall incidence rate of 137 cases and 3.7 deaths per 100,000 people.[2] With just 15% of the population of 25 million fully vaccinated by the end of July 2021, stay-at-home orders ('lockdown') were the country's main measure of disease control.[3] Families caring for children and young people (referred to as children hereafter) were substantially affected by lockdown,[4 5] required to juggle working from home, responsibility for their children's remote learning and care during school and childcare closures, with minimal social opportunities due to closure of sports and play centres, and reduced access to healthcare. Despite mental health having "greater impact on human activity than any other non-communicable illness",[4] it appeared to be given little consideration in the majority of Australia's lockdown policies. With future variants and pandemics likely, Australia's experience offers insight into the mental health experiences of lockdown, effectively without the compounding direct harms of the virus.

The evidence for the indirect and longer-term impacts of the pandemic is quickly evolving. In a review of the global mental health evidence from the first year (to April 2021), Aknin et al., reported a peak in adults' psychological distress in the early months.[4] While many studies reported a decline to pre-pandemic levels by mid-2020,[4] the authors found that mental health inequities were sustained or exacerbated for adults who were younger, female, child-rearing, or with fewer socioeconomic resources.[4 5] However, published data on the specific experience of parents (termed caregivers throughout) are scarce. Evidence reviews of children's experiences also suggest that their mental health declined during the pandemic.[6 7] In Racine and colleagues' (2021) meta-analysis of 29 studies published in the first year of the pandemic, prevalence estimates for depression and anxiety in

https://mc.manuscriptcentral.com/bmjpo

Confidential: For Review Only

children doubled, and were higher over time, and for older adolescents and females.[8] However, the available systematic reviews are limited by over-representation of data from the early months of the pandemic,[6-8] and previous pandemics show that negative mental health effects can persist.[9] As such, there are urgent calls for research into the "long-term mental health consequences of living through the pandemic",[4] which prioritises children.[5]

In response to these calls, we aimed to describe the mental health experiences of caregivers and children during the pandemic, using Australia's only nationally representative, repeated cross-sectional survey of families with children. Unlike many high-income countries, Australia's low incidence of COVID-19 over the first eighteen months of the pandemic makes it possible to examine the mental health experiences in the relative absence of the disease. Based on the gender, age and socioeconomic disparities identified in the published evidence reviews,[4 8] we hypothesised that caregiver mental health and perceived impacts of the pandemic on caregiver and child mental health would be worse: (i) for caregivers who identified as female compared with male, and older compared with younger children, (ii) for families experiencing greater socioeconomic adversity compared with more socioeconomic advantage and (iii) with increasing total length of lockdown.

Confidential: For Review Only

METHODS

Design and procedure: The Royal Children's Hospital (RCH) National Child Health Poll comprises periodic cross-sectional surveys of approximately 2000 Australian caregivers of children aged 0-17 years. Data collection is contracted to the Online Research Unit who obtain written informed consent and draw a nationally representative sample of caregivers using stratified random sampling from their panel of over 350,000 adults aged 18 years or older, who live in Australia and have internet access. Surveys are administered in English, with a reading level equivalent to sixth grade (the end of elementary school). Responses are anonymous, and respondents are remunerated with points exchangeable for department store gift vouchers.

Questions about mental health – the focus of the current study – were introduced after COVID-19 began and were collected in three surveys. The dates of data collection were: (1) 15-23 June 2020, after a first national lockdown (March-May 2020) eased; (2) 15-29 September 2020, when only metropolitan residents of Victoria were in a second, stricter lockdown (July-November 2020); and (3) 20-29 July 2021, when multiple states/territories were in and out of lockdown (June 2021 onwards)). The RCH Human Research Ethics Committee approved this research (February 2020, #35254).

Patient and Public Involvement: The research questions and design of this study were informed by previous RCH Poll surveys, which asked caregivers to identify the child health issues of most concern to them and which child health topics should be included in future polls. At the end of each survey, participants were informed of the study website where all research reports are accessible to the public. As each survey is collected from a cross-sectional, population-based online survey of a random sample, respondents were not directly involved in the recruitment or conduct of the study.

Confidential: For Review Only

Measures: Each survey collected the demographic, socioeconomic and pandemic-related financial characteristics described in Table 1. To achieve high response rates and population representativeness, the surveys are intentionally brief and ask questions that are simple in their phrasing and response options. Table 1 also describes how the two measures of lockdown (current and total length) were defined according to Australia's state and federal government pandemic responses. Families were assigned the Australian Bureau of Statistics' (ABS) Socio-Economic Indexes for Areas (SEIFA) Index of Relative Disadvantage, a national area level index derived from census data for all individuals living in a postcode, with higher scores indicating greater advantage. Caregivers self-reported their mental health (Kessler-6), and the perceived impact of the pandemic on their own mental health, and the mental health of each child in their care (details in Table 1).

Analysis: To reduce effects of non-response and non-coverage and therefore approximate the population distributions of mental health experiences, the mental health measures were weighted using ABS distributions of caregiver age, gender, family structure (sole-caregiving, number of children and any under 5 years), state/territory and SEIFA. As the primary aim of this study was to describe the mental health experiences of Australian families during the pandemic, we calculated simple weighted proportions of the mental health measures for each survey. These were described overall and by demographic, socioeconomic and pandemic-related financial and lockdown characteristics.

To investigate the specific relationship between lockdown and mental health, we used generalised linear models to calculate the association between each mental health measure and time of the survey, according to state/territory grouping (as a proxy for total length of lockdown, see Table 1). The binomial models investigated whether increasing length of lockdown increased the frequency of negative mental health experiences over time, after adjusting for caregiver gender, sole caregiver

Confidential: For Review Only

status, education, SEIFA, and cultural or linguistic diversity (measures described in Table 1). The child mental health model was additionally adjusted for poor caregiver mental health (Kessler-6, see Table 1) and clustering at the level of family. The estimated models were then used to predict probabilities of each mental health outcome, by state/territory grouping, at the three survey timepoints, adjusted for the covariates in the model.

Confidential: For Review Only

Table 1. Demographic, socioeconomic, lockdown and mental health measures

**Measure**	**Description**
Demographic	
Age	Collected for caregivers and children, reported in years. Families and children were differentially affected by lockdown depending on whether children were attending early education centres (day-care) or school. This is because schools and early education centres were closed for different, and varying periods of the pandemic. Caregivers of school aged children were required to facilitate home learning, which was arguably more difficult for caregivers of young (compared with older) children. Similarly, families were required to care for young children when early education centres closed, which made it difficult to work from home and also balance other responsibilities. Child age was used as a proxy for these experiences, and categorised to represent pre-school (0-4 years), primary/elementary school (5-11 years) and high school (12-17 years).
Gender	Collected for caregivers and children: response options “male”, “female”, “other”. Two caregivers and no children identified as "other". Before the 2020 Australian census, "other" gender data were not collected for the Australian population, so it was not possible to calculate weights for these caregivers. Hence, they were dropped from the analysis, and the gender variable was dichotomised into “female” compared with “male”.
Sole caregiver	Question “Are you the sole (single) parent or carer of a child 17 years of age or younger?”, binary response options “yes” (one-caregiver household) compared with “no” (multi-caregiver household).
Caregiver education	Question “What is the highest level of schooling / education you have completed?”. Responses were trichotomised into three categories that meaningfully represented education as a socioeconomic measure for Australians: (1) “Year 12 or less” (response options: less than year 10, Year 10 or equivalent (e.g. school certificate), Year 12 or equivalent); (2) “vocational training certificate” (response options: trade/apprenticeship (e.g. carpenter), certificate/diploma (e.g. Cert IV Childcare)); or (3) “university degree” (response options: undergraduate university degree, postgraduate university degree (e.g. Masters, Doctorate, PhD).
Caregiver country of birth	Question “Were you born in Australia”, binary response options “yes” (born in Australia) compared with “no” (outside Australia).
Home language	Question “Do you speak a language other than English at home?”, binary response options “yes” (other than English) compared with “no” (English).
Cultural or linguistic diversity	A composite of the two above variables, caregiver country of birth and home language, to represent respondents who answered “yes” to either or both items, compared with “no” (answering no to both items).
Socioeconomic and lockdown	
Job/income loss during COVID-19	Three items drawn from the CoRonavIruS Health Impact Survey (CRISIS) caregiver version:[10] “What changes in employment or income have occurred in your household due to coronavirus/COVID-19?” (binary response options “yes” compared with “no”) including: “job loss by one caregiver”; “job loss by two caregivers” and “reduced total household income”. A binary variable describing any job loss (by one or two caregivers) or reduction in income due to COVID-19 (compared with not) was created.
Low income (<$1000)	A binary variable based on current total household income before tax, categorised into 10 options ranging from “less than $500 p/week” to “more than $3,000 p/week”, plus “prefer not to say”. In 2021, to protect against the economic fallout of lockdown, the Australian federal government rapidly implemented a suite of short-term financial supports, which included an unemployment supplement (‘JobSeeker’) which doubled recipients’ social welfare benefits from $550 to $1,100 a fortnight, and a wage supplement for eligible businesses to retain their workforce (‘JobKeeper’). These social policy changes represented some of the largest (albeit temporary) in Australia’s history and were so significant that, by September 2020, levels of poverty and housing stress in Australia were substantially lower than the levels directly preceding COVID-19. To capture any relationships between income poverty and mental health, we created a binary variable summarising low income (“less than AU$1,000 p/week” compared with more) based on Australian thresholds.[11] n=767 caregivers preferred not to report income, so this variable should be interpreted with caution.

Confidential: For Review Only

Couldn't afford essential items	Eight items adapted from the Household, Income and Labour Dynamics in Australia (HILDA) Survey Wave 18 Household Questionnaire Material Deprivation Module [12] asking "In the last month, because of money pressure did you miss or put off" (binary response options: "yes" compared with "no"): mortgage or rent repayments; electricity, gas, water bills; food; healthcare; prescription medicines; home or car insurance; mobile phone bills; and internet. A binary summary variable was created to denote "any material deprivation" inability to pay for one or more essential items compared with "none".
Current lockdown	All Australian states and territories experienced a first national, 10-week lockdown from 23 March to 1 June 2020. Victoria experienced a second extended and more severe, 20-week lockdown from 8 July-23 November 2020. There were several short (less than a week) lockdowns in the intervening months across states, before New South Wales experienced its second extended period of lockdown, which began incrementally on 26 June 2021. Victoria experienced its fifth (in total) lockdown from 16-27 July 2021, before an extended lockdown subsequently began a week later on 5 August 2021. Current lockdown was therefore categorised as follows:<br>• The national lockdown had eased for the collection of the June 2020 Poll, so no (denoted 'N' in Tables 2-3) Australians were in lockdown. Thus, the number/proportion not in lockdown represents the whole cohort (and is equal to the 'overall' numbers/proportions at top of Tables 2-3).<br>• For the September 2020 Poll, only residents of metropolitan Melbourne in the state of Victoria were in lockdown (denoted 'Y' for "yes") compared with all other Australians (N). This was categorised according to respondents who reported living in Victoria and living in "metropolitan" compared with "regional/rural" areas.<br>• By July 2021, many states/territories were going in and out of lockdown, and this question was added into the Poll and reported on directly with the question "are you currently under stay-at-home orders or restrictions due to COVID-19 (also known as 'lockdown')?", responses "yes" compared with "no".
Total length of lockdown	Trichotomous variable based on total length of lockdown experienced by each state/territory. By 31 July 2021, the total length of COVID-19 lockdown was greatest for the state of Victoria ("Vic", total 31 weeks); followed by the state of New South Wales ("NSW", total 15 weeks) and then all "other" states and territories (total range 10-12 weeks). The following geographical categories were used as a proxy for total length of lockdown: (1) Victorian (most), (2) NSW, and (3) Other (least).
Mental health	
Caregiver mental health	6 items of the Kessler-6 (K6) assessing caregivers' self-reported anxiety and depressive symptoms encountered in the last 4 weeks. Scored on a 5-point Likert scale from 1 "none of the time" to 5 "all of the time". Summarised into (a) a continuous total score, and (b) a binary variable indicating "poor mental health" (total score 19 or more) compared with not (total score 6-18).[13]
Perceived impact of the pandemic on mental health	A 5-point item adapted from UK Young Minds Matter Study,[14] describing the perceived impact of COVID-19 on mental health, dichotomised into negative ("small negative/large negative") compared with positive ("none/small positive/large positive"). Reported by caregivers for (a) themselves and (b) each child.

Confidential: For Review Only

RESULTS

In June 2020, n=2020 of 2697 (74.9%) caregivers approached provided data for themselves and 3411 children. In September 2020, 1434/1769 (81.1%) caregivers provided data for 2553 children. In July 2021, 2508/3925 (63.9%) provided data for 4327 children. Table 2 presents the sample characteristics for each survey. The SEIFA quintiles suggested strong response bias towards more advantaged groups. There were also some differences between surveys in characteristics such as the proportion of respondents caring for young children, caregiver gender, sole caregiving, and SEIFA; characteristics that were used to create the sample weights (see *Analysis*).

For the three surveys overall, children's mean age was 9.3 years (standard deviation (SD) 5.0 years) and 47.8% were female. Caregivers' mean age was 42.2 years (SD 9.1 years), 54.0% were female, and each caregiver cared for 1.7 children on average (SD 0.8). One-quarter were sole caregivers, 56.6% had a university degree, 25.6% were born outside Australia, and 22.4% spoke a language other than English at home. Twenty-eight percent of caregivers reported job/income loss due to COVID-19; 17.2% reported low household income and 29.3% were unable to afford at least one essential item in the month prior. The proportions of respondents in lockdown were 0% (June 2020), 29.2% (September 2020) and 56.5% (July 2021).

Table 3 presents the weighted mental health experiences for each survey, separately by characteristics. When considering patterns over time, the proportions of respondents who reported poor mental health according to the K6 was similar between surveys. Perceived impacts on caregiver mental health became more frequently negative between June 2020 and July 2021, although there were no consistent patterns between June 2020 and September 2020, or between September 2020 and July 2021. In contrast, there was a clear pattern that

Confidential: For Review Only

perceived impacts on children's mental health became more frequently negative across the three waves of data collection.

When considering differences between subgroups, perceived negative impacts on mental health were more common for caregivers and children when reported by female than male caregivers, and for older compared with younger children, but were similar between child genders. Poor mental health (K6) was more common for groups typically experiencing more social adversity, such as sole caregiving compared with multi-caregiver households, having a home language other than English (but not for country of birth), and several financial and lockdown characteristics (lower SEIFA and income, couldn't afford essentials, and increasing total or current lockdown). In contrast with the K6, perceived impacts of the pandemic on caregiver and child mental health were more frequently negative for families experiencing greater social advantage, such as multi-caregiver (compared with sole caregiver) households and higher (compared with lower) education, SEIFA, and income. Job or income loss, and increasing total length of lockdown, were associated with increasing negative mental health experiences across all three measures, across the three timepoints.

Figure 1 shows that the three measures of negative mental health experiences increased for Victorian and NSW families by July 2021 compared with June 2020. Increases in the proportions of caregivers reporting poor mental health (K6) aligned with the second extended lockdowns; for Victorians this was September 2020 and for NSW it was July 2021 (Figure 1(a), Supplementary Table 1(a)). In contrast, there was weak evidence of change in the proportions of caregivers in Other states/territories reporting poor mental health (K6) over time. The proportions of caregivers and children who perceived negative impacts of the pandemic on their mental health increased from June 2020 to July 2021 (Figure 1(b-c), Supplementary Tables 1(b-c)). Increased proportions of Victorians perceived negative impacts on their mental health in September 2020 and again in July 2021. More caregivers

Confidential: For Review Only

from NSW and the Other states/territories self-reported negative impacts in July 2021 compared with June or September 2020.

Confidential: For Review Only

Table 2. Sample characteristics by survey, described with the number of respondents (proportion).

		Number of respondents (%)		
**Characteristic**	**Subgroup**	**June 2020**	**Sept 2020**	**July 2021**
Child		**N=3411**	**N=2553**	**N=4327**
Age in years	0-4	628 (18.4)	520 (20.4)	1291 (29.8)
	5-11	1356 (39.8)	1153 (45.2)	1705 (39.4)
	12-17	1427 (41.8)	880 (34.5)	1331 (30.8)
Gender	F	1664 (48.7)	1242 (48.7)	2013 (46.5)
	M	1747 (51.2)	1311 (51.4)	2314 (53.5)
Caregiver		**N=2020**	**N=1434**	**N=2508**
Gender	F	1003 (49.7)	758 (52.9)	1458 (58.1)
	M	1017 (50.4)	676 (47.1)	1050 (41.9)
Sole carer	Yes	462 (22.9)	282 (19.7)	704 (28.1)
	No	1558 (77.1)	1152 (80.3)	1804 (71.9)
Education [a]	Y12	326 (16.1)	239 (16.7)	422 (16.8)
	Cert.	518 (25.6)	390 (27.2)	693 (27.6)
	Uni.	1176 (58.2)	805 (56.1)	1393 (55.5)
Born outside Australia	Yes	546 (27.4)	329 (23.4)	622 (25.4)
	No	1444 (72.6)	1078 (76.6)	1827 (74.6)
Home language other than English	Yes	413 (20.5)	324 (22.6)	600 (23.9)
	No	1607 (79.6)	1110 (77.4)	1908 (76.1)
Socio-economic				
SEIFA quintile (1=most disadvantage, 5=least disadvantage)	1	213 (10.6)	172 (12.0)	327 (13.0)
	2	273 (13.5)	245 (17.1)	391 (15.6)
	3	370 (18.3)	250 (17.4)	511 (20.4)
	4	457 (22.7)	319 (22.3)	571 (22.8)
	5	705 (34.9)	448 (31.2)	707 (28.2)
Low income (<$1000) [c]	Yes	254 (14.7)	212 (16.9)	427 (19.2)
	No	1480 (85.3)	1040 (83.1)	1782 (80.7)
Couldn't afford essential items [d]	Yes	527 (26.1)	400 (27.9)	822 (32.8)
	No	1493 (73.9)	1034 (72.1)	1686 (67.2)
Pandemic experience				
Job/income loss [b]	Yes	559 (27.7)	381 (26.6)	711 (28.4)
	No	1461 (72.3)	1053 (73.4)	1797 (71.7)
Current lockdown [e]	Yes	0 (0)	419 (29.2)	1416 (56.5)
	No	2020 (100)	1015 (70.8)	1092 (43.5)
State as a proxy for total length of lockdown (Vic=most, Other=least) [e]	Vic	612 (30.3)	473 (33.0)	746 (29.7)
	NSW	617 (30.5)	449 (31.3)	791 (31.5)
	Other	791 (39.2)	512 (35.7)	971 (38.7)

Vic: Victoria, NSW: New South Wales. Data were weighted using national demographic distributions for caregiver age, gender, family structure (sole-caregiving, number of children and any under 5 years), state/territory and Socio-Economic Indexes for Areas Index of Relative Disadvantage (SEIFA).

[a] Highest education coded as Year 12 or less (up to the end of high/secondary school; Y12); vocational training certificate ("cert."); or university degree ("uni").

[b] Job loss by one or two adults, or reduction in income, due to COVID-19.

[c] Low-income defined according to Australian definitions of income-poverty. Missing 767 caregivers who preferred not to report income.

[d] Any one or more of mortgage or rent; electricity, gas, water bills; food; healthcare; prescription medicines; home or car insurance; mobile phone bills; internet, in the last month.

[e] No Australians were in lockdown for the June 2020 Poll, so the number/proportion is for the whole cohort. For the September 2020 Poll, only residents of metropolitan Melbourne in the state of Victoria were in lockdown (Y) compared with all other Australians (N). In July 2021, many states/territories were going in and out of

Confidential: For Review Only

lockdown, and this question was asked directly (Y/N). Overall, Victorians experienced the longest total lockdown, followed by NSW, followed by Other states and territories.

Confidential: For Review Only

Table 3. Mental health experiences by sample characteristic and survey, described with the number of respondents (weighted proportion).

		Poor caregiver mental health (K6) [f]			Perceived negative impact on caregiver mental health [g]			Perceived negative impact on child mental health [g]		
**Characteristic**	**Subgroup**	**June 2020 N=2020**	**Sept 2020 N=1434**	**July 2021 N=2508**	**June 2020 N=2020**	**Sept 2020 N=1434**	**July 2021 N=2508**	**June 2020 N=3411**	**Sept 2020 N=2553**	**July 2021 N=4327**
Overall	All	264 (17.4)	247 (19.5)	541 (18.9)	949 (47.4)	708 (50.3)	1490 (60.0)	1055 (25.6)	884 (33.1)	1807 (44.0)
Child										
Age in years	0-4	-	-	-	-	-	-	81 (12.8)	101 (19.9)	330 (24.2)
	5-12	-	-	-	-	-	-	440 (32.3)	414 (37.3)	789 (46.9)
	13-17	-	-	-	-	-	-	534 (38.2)	369 (43.7)	688 (51.5)
Gender	F	-	-	-	-	-	-	525 (25.1)	438 (32.8)	876 (45.5)
	M	-	-	-	-	-	-	530 (26.0)	446 (33.4)	931 (42.7)
Caregiver										
Gender	F	153 (18.3)	110 (18.0)	338 (20.5)	489 (49.6)	410 (53.9)	920 (63.7)	512 (25.9)	521 (35.1)	1099 (45.7)
	M	111 (15.8)	137 (21.8)	203 (16.6)	460 (44.1)	298 (44.7)	570 (54.7)	543 (25.1)	363 (30.0)	708 (41.6)
Sole carer	Yes	137 (36.1)	100 (36.5)	268 (32.6)	199 (42.7)	99 (36.2)	335 (44.3)	190 (24.3)	144 (27.1)	397 (30.9)
	No	127 (10.2)	147 (14.8)	273 (15.0)	750 (49.2)	609 (54.1)	1155 (64.5)	865 (26.0)	740 (34.8)	1410 (47.9)
Education [a]	Y12	55 (21.7)	61 (26.9)	124 (15.8)	141 (40.2)	98 (42.9)	211 (47.3)	148 (21.2)	115 (24.3)	293 (39.2)
	Cert.	73 (21.4)	65 (19.9)	152 (20.0)	244 (48.7)	204 (56.1)	418 (64.6)	293 (28.6)	264 (34.9)	524 (51.1)
	Uni.	136 (15.8)	121 (16.9)	265 (17.5)	564 (48.8)	406 (49.6)	861 (61.5)	614 (25.3)	505 (34.9)	990 (41.8)
Born outside Australia	Yes	58 (12.5)	40 (11.7)	124 (16.9)	243 (42.4)	169 (49.2)	379 (60.0)	253 (23.0)	184 (32.0)	396 (40.2)
	No	198 (18.9)	198 (21.0)	405 (19.6)	694 (49.4)	530 (50.8)	1080 (60.1)	789 (26.8)	695 (33.7)	1375 (45.3)
Home language other than English	Yes	80 (20.9)	79 (22.5)	176 (25.1)	172 (40.6)	154 (47.6)	337 (58.9)	178 (21.7)	181 (31.7)	352 (39.5)
	No	184 (16.3)	168 (18.6)	365 (17.0)	777 (49.4)	554 (51.1)	1153 (58.9)	877 (26.8)	703 (33.5)	1455 (45.4)
Socio-economic										
SEIFA quintile (1=most disadvantage, 5=least disadvantage)	1	41 (22.1)	35 (20.7)	97 (26.1)	86 (41.4)	77 (47.4)	172 (56.0)	115 (27.1)	90 (31.3)	223 (42.0)
	2	50 (20.5)	53 (25.6)	99 (21.4)	125 (46.1)	117 (51.3)	215 (60.6)	133 (21.1)	157 (32.6)	262 (45.1)
	3	49 (16.2)	41 (19.3)	114 (17.9)	186 (53.1)	129 (51.9)	295 (58.7)	191 (27.3)	170 (34.5)	350 (40.8)
	4	66 (17.9)	54 (20.8)	108 (16.0)	205 (44.1)	149 (49.6)	350 (62.4)	242 (26.6)	172 (31.0)	392 (42.8)
	5	58 (9.4)	64 (11.1)	123 (16.5)	347 (51.7)	236 (51.0)	458 (67.1)	374 (26.3)	295 (35.7)	580 (52.9)
Low income (<$1000) [c]	Yes	69 (27.5)	58 (31.3)	171 (29.5)	120 (53.4)	89 (45.6)	230 (51.2)	111 (26.1)	133 (33.3)	271 (35.3)

Confidential: For Review Only

		Poor caregiver mental health (K6) [f]			Perceived negative impact on caregiver mental health [g]			Perceived negative impact on child mental health [g]		
**Characteristic**	**Subgroup**	**June 2020 N=2020**	**Sept 2020 N=1434**	**July 2021 N=2508**	**June 2020 N=2020**	**Sept 2020 N=1434**	**July 2021 N=2508**	**June 2020 N=3411**	**Sept 2020 N=2553**	**July 2021 N=4327**
	No	165 (15.5)	167 (17.3)	326 (16.6)	686 (45.2)	507 (49.2)	1064 (61.0)	774 (24.8)	631 (32.4)	1292 (44.6)
Couldn't afford essential items [d]	Yes	359 (37.4)	156 (38.4)	341 (35.5)	263 (50.3)	165 (44.8)	469 (59.2)	270 (26.5)	236 (33.9)	586 (42.6)
	No	96 (8.8)	91 (12.3)	200 (11.2)	686 (46.2)	543 (52.4)	1021 (60.4)	785 (25.1)	648 (32.8)	1221 (44.7)
Pandemic experience										
Job/income loss [b]	Yes	96 (23.7)	93 (27.5)	218 (28.8)	306 (51.0)	224 (59.5)	486 (71.1)	305 (25.0)	297 (40.7)	613 (55.8)
	No	168 (15.1)	288 (16.4)	323 (15.3)	643 (46.1)	484 (46.8)	1004 (55.9)	750 (25.7)	587 (30.2)	1194 (39.7)
Current lockdown [e]	Yes	n/a	83 (24.0)	353 (23.5)	n/a	240 (58.1)	867 (62.9)	n/a	355 (44.1)	1114 (49.6)
	No	264 (17.4)	164 (17.7)	188 (13.3)	949 (47.4)	468 (47.3)	623 (56.4)	1055 (25.6)	529 (28.7)	693 (37.3)
State as a proxy for total length of lockdown (Vic=most, Other=least) [e]	Vic	85 (17.7)	93 (23.3)	200 (25.1)	284 (48.9)	266 (57.5)	483 (66.4)	321 (23.8)	389 (42.5)	626 (51.4)
	NSW	96 (21.6)	78 (18.6)	188 (22.0)	307 (49.2)	213 (49.0)	477 (60.8)	306 (25.9)	267 (34.4)	603 (48.9)
	Other	83 (12.4)	76 (16.6)	153 (11.2)	358 (43.9)	229 (44.1)	530 (54.4)	428 (26.9)	228 (21.0)	578 (33.7)

Vic: Victoria, NSW: New South Wales. proportions were weighted using national demographic distributions for caregiver age, gender, family structure (sole-caregiving, number of children and any under 5 years), state/territory and Socio-Economic Indexes for Areas Index of Relative Disadvantage (SEIFA). .

[a] Highest education coded as Year 12 or less (up to the end of high/secondary school; Y12); vocational training certificate ("cert."); or university degree ("uni").

[b] Job loss by one or two adults, or reduction in income, due to COVID-19.

[c] Low-income defined according to Australian definitions of income-poverty. Missing 767 caregivers who preferred not to report income.

[d] Any one or more of mortgage or rent; electricity, gas, water bills; food; healthcare; prescription medicines; home or car insurance; mobile phone bills; internet, in the last month.

[e] No Australians were in lockdown for the June 2020 Poll, so the proportion is for the whole cohort (see 'Overall' at top of Table). For the September 2020 Poll, only residents of metropolitan Melbourne in the state of Victoria were in lockdown (Y) compared with all other Australians (N). In July 2021, many states/territories were going in and out of lockdown, and this question was asked directly (Y/N). Overall, Victorians experienced the longest total lockdown, followed by NSW, followed by Other states and territories.

[f] Kessler-6 (K6) dichotomised into a binary "poor mental health" (total score 19 or more) compared with not (total score 6-18).

[g] Dichotomised into negative ("small negative/large negative") compared with positive ("none/small positive/large positive").

Confidential: For Review Only

DISCUSSION

This study describes the mental health experiences captured by only nationally representative and repeated cross-sectional survey of Australian families over 12 months of the COVID-19 pandemic. Poor caregiver mental health (K6) was stable and similar between male and female genders. In contrast, the proportion of caregivers reporting a negative impact on mental health increased over time, and was more common for female caregivers, and older (compared with younger) children. Across the three surveys, job or income loss due to the pandemic was associated with poorer caregiver mental health and perceived negative impacts on caregiver and child mental health. While poor caregiver mental health was more common for families experiencing greater social adversity, perceived impacts on mental health were more frequently negative for the more socially advantaged groups. Caregivers in states/territories who experienced the least total lockdown reported similar K6 scores over time. Otherwise, poor mental health and perceived negative impacts on mental health increased over the course of the three surveys and with increasing total length lockdown.

A striking finding of this study is that caregiver mental health (K6) was stable over time (except as related to total length of lockdown), while the perceived impact of the pandemic on the mental health of caregivers and children was increasingly negative. This highlights the variation in mental health experiences and measures. The K6 offers a clinical measure of mental health symptomatology, while the other is a relative measure that considers perceived impact over time. These complementary measures offer different insights into the mental health experiences of families during the pandemic. On one hand, the absolute numbers of caregivers experiencing poor mental health were similar between surveys. On the other hand, caregivers were perceiving negative impacts, which are real and concerning.

Confidential: For Review Only

Comparable data on the mental health experiences specific to caregivers of children during the pandemic are limited. Aknin and colleagues' review of mental health experiences during COVID-19 suggest that levels of psychological distress in general adult populations may have declined from an early peak in the first months of the pandemic.[4] However, the Pulse of the Nation (TPTN), a weekly, cross-sectional representative survey of Australian adults throughout the pandemic,[15] found that mental distress (measured with a single item that highly correlated with the K6) increased during 2020. The authors described this as driven by increasing financial stress.[15] This aligns with our finding that job or income loss during the pandemic was associated with worse mental health experiences.

In our study, length of lockdown was clearly associated with negative mental health experiences. While the RCH Poll lacked pre-pandemic data, substantially more caregivers reported poor mental health on the K6 (average 18%) in the Polls throughout the pandemic than representative Australian adult data collected pre-pandemic (8% in 2017) or during the first national lockdown (11%).[16] Notably, TPTN data found that increases in mental distress were highest for parents, tripling from 8% pre-pandemic to 24% during the pandemic.[15] Our finding of increasing perceived negative impacts of the pandemic on children's mental health are consistent with the emerging reviews of the published evidence.[9]

Strengths of our study included the large cross-sectional and nationally representative surveys, which employed strong methodology (surveys piloted and included the validated K6); collected data on caregiver and child mental health; surveyed female and male caregivers; and achieved good response rates. In other Polls, indicators (frequency/prevalence) across a range of topics are almost universally consistent with more traditionally obtained estimates, providing support for the sample selection and survey administration methods. By July 2021, we found that perceived impacts of the pandemic on

https://mc.manuscriptcentral.com/bmjpo

Confidential: For Review Only

mental health were negative for half of children and two-thirds of adults. This highlights how general pandemic stress, and disruption to family life including employment, routines, and social interactions, can adversely affect caregivers and children. Despite Australia's low incidence of COVID-19, the increases in psychological distress are consistent with data from countries with high infection rates and the increases in distress are sizable.[4 6 8] This pattern has been described for previous health crises; following the H1N1 pandemic, a quarter of parents and 30% of children who were required to isolate met criteria for PTSD symptoms.[17]

This study also had limitations. The reliance on caregiver-report, from only one caregiver per household, means the child rating may be biased by caregiver perception. We did not collect a validated measure of children's mental health that would provide a measure of clinical impact. The finding of different social gradients for the K6 compared with the negative impact items may represent differing expectations and emphasises the need for validated measures for anticipating the longer-term consequences of lockdown, which ideally would be collected from all caregivers and children directly. The RCH Poll only samples adults who are 18 years and older, so younger caregivers are missing, and a significant proportion of caregivers did not report family income. However, the weighted and adjusted analyses support the generalisability of findings to the broader population of Australian families raising children and young people.

Australia's reliance on lockdown has meant that caregivers had to concurrently parent, work, and facilitate remote schooling. Our findings show that, despite the low disease incidence, the pandemic experience has undermined caregivers' and children's mental health. Understanding specific risk factors for poorer mental health over time was beyond the scope of the cross-sectional data. However, our findings support the inequities evident in existing research. As researchers have suggested,[4 6 8] understanding and prioritising population risk

Confidential: For Review Only

groups should be the subject of ongoing research. Clearly, pandemic response and recovery planning must prioritise and incorporate mental health supports. To this end, we endorse the recommendations proposed by Aknin and colleagues.[6] These include prioritising safe access to childcare and schools; greater investment in evidence-informed mental health services including telehealth, and its integration with social care systems; and mental health promotion across communities such as work and schools. The authors also emphasise the importance of monitoring mental health. As our findings demonstrate, mental health is changing during the pandemic. Understanding families' experiences is necessary to inform policy effort with a greater level of precision, so that we can respond to the evolving mental health needs of children and their families.

Confidential: For Review Only

FIGURE LEGEND

Figure 1. Estimated probabilities over time (by survey) and by state/territory (as a proxy for total length of lockdown) adjusted for potential confounders for (a) caregiver K6, and negative mental health impacts for the (b) caregiver and (c) children.

ACKNOWLEDGMENTS AND FUNDING

We thank all families who took part in the Royal Children's Hospital National Child Health Polls. The National Child Health Polls are funded by The Royal Children's Hospital Foundation (Grant #n/a). This work was supported by The Healthier Wealthier Families project, which was supported by the Helen Macpherson Smith Trust Impact Grant #9523 and The Corella Fund (Grant #n/a). The Murdoch Children's Research Institute (MCRI) administered research grants for the work and provided infrastructural support (as study sponsor) to its staff but played no role in the conduct or analysis of the research. Research at the MCRI is supported by the Victorian Government's Operational Infrastructure Support Program. Author AP was supported by The Erdi Foundation Child Health Equity (COVID-19) Scholarship (Grant# n/a). Author SG was supported by a NHMRC Practitioner Fellowship (#1155290).

COMPETING INTERESTS

None to declare.

AUTHOR CONTRIBUTIONS

Anna Price: Conceptualisation, Validation, Formal analysis, Investigation, Resources, Data curation, Writing – Original Draft, Writing - Review & Editing, Visualisation, Supervision, Project administration, Funding acquisition.

Mary-Anne Measey: Conceptualisation, Methodology, Validation, Investigation, Data curation, Writing - Review & Editing.

Monsurul Hoq: Methodology, Validation, Formal analysis, Investigation, Data curation, Writing - Review & Editing.

Anthea Rhodes: Conceptualisation, Methodology, Investigation, Resources, Writing - Review & Editing, Supervision, Project administration, Funding acquisition.

Sharon Goldfeld: Conceptualisation, Methodology, Investigation, Resources, Writing - Review & Editing, Supervision, Funding acquisition.

AR and SG share senior authorship.

DATA SHARING STATEMENT

The data underlying the results presented in the study are available from the RCH Child Health Poll, please contact child.healthpoll@rch.org.au.

Confidential: For Review Only

REFERENCES

1. Hale T, Angrist, Noam. , Goldszmidt R, Kira B, Petherick A, Phillips T, et al. A global panel database of pandemic policies (Oxford COVID-19 Government Response Tracker). Nature Human Behaviour. https://doi.org/10.1038/s41562-021-01079-8. Retrieved from: https://ourworldindata.org/grapher/covid-stringency-index [Online Resource] (2021). 2021.
2. Australian Department of Health. Coronavirus (COVID-19) at a glance - 31 July 2021. https://www.health.gov.au/sites/default/files/documents/2021/08/coronavirus-covid-19-at-a-glance-31-july-2021_0.pdf.
3. Mathieu E, Ritchie H, Ortiz-Ospina E, et al. A global database of COVID-19 vaccinations. *Nature human behaviour* 2021:1-7.
4. Aknin L, De Neve J-E, Dunn E, et al. Mental health during the first year of the COVID-19 pandemic: A review and recommendations for moving forward. *Perspectives on Psychological Science* 2021
5. Centre for Mental Health. Addressing inequities in mental health research exacerbated by Covid-19. A report by the Mental Health Research Group. 6 July 2021 [https://www.centreformentalhealth.org.uk/publications/fit-for-purpose, last accessed 24 Nov 2021].
6. Samji H, Wu J, Ladak A, et al. Review: Mental health impacts of the COVID-19 pandemic on children and youth – a systematic review. *Child and Adolescent Mental Health*;n/a(n/a) doi: https://doi.org/10.1111/camh.12501
7. Jones EAK, Mitra AK, Bhuiyan AR. Impact of COVID-19 on Mental Health in Adolescents: A Systematic Review. *Int J Environ Res Public Health* 2021;18(5) doi: 10.3390/ijerph18052470 [published Online First: 2021/04/04]
8. Racine N, McArthur BA, Cooke JE, et al. Global Prevalence of Depressive and Anxiety Symptoms in Children and Adolescents During COVID-19: A Meta-analysis. *JAMA Pediatrics* 2021;175(11):1142-50. doi: 10.1001/jamapediatrics.2021.2482
9. Meherali S, Punjani N, Louie-Poon S, et al. Mental Health of Children and Adolescents Amidst COVID-19 and Past Pandemics: A Rapid Systematic Review. *International Journal of Environmental Research and Public Health* 2021;18(7):3432.
10. Nikolaidis A, Paksarian D, Alexander L, et al. The Coronavirus Health and Impact Survey (CRISIS) reveals reproducible correlates of pandemic-related mood states across the Atlantic. *Sci Rep* 2021; 11(1). (accessed 2021/04//).
11. Davidson P, Saunders P, Bradbury B, et al. Poverty in Australia 2020: Part 1, Overview. ACOSS/UNSW Poverty and Inequality Partnership Report No. 3, Sydney: ACOSS.; 2020.
12. Wilkins R, Lass I. The Household, Income and Labour Dynamics in Australia Survey: Selected Findings from Waves 1 to 16. Melbourne Institute: Applied Economic & Social Research, University of Melbourne., 2018.
13. Furukawa TA, Kessler RC, Slade T, et al. The performance of the K6 and K10 screening scales for psychological distress in the Australian National Survey of Mental Health and Well-Being. *Psychol Med* 2003;33(2):357-62. doi: 10.1017/s0033291702006700 [published Online First: 2003/03/08]
14. Young Minds. Coronavirus: Impact on young people with mental health needs. https://youngminds.org.uk/media/3708/coronavirus-report_march2020.pdf. 2020.
15. Broadway B, Payne A, A, Salamanca N. Coping with COVID-19: Rethinking Australia. Melbourne Institute: Applied Economic & Social Research, the University of Melbourne., 2020.
16. Edwards B, Biddle N, Gray M, et al. Initial impacts of COVID-19 on mental health in Australia. 2020
17. Sprang G, Silman M. Posttraumatic stress disorder in parents and youth after health-related disasters. *Disaster medicine and public health preparedness* 2013;7(1):105-10.

https://mc.manuscriptcentral.com/bmjpo

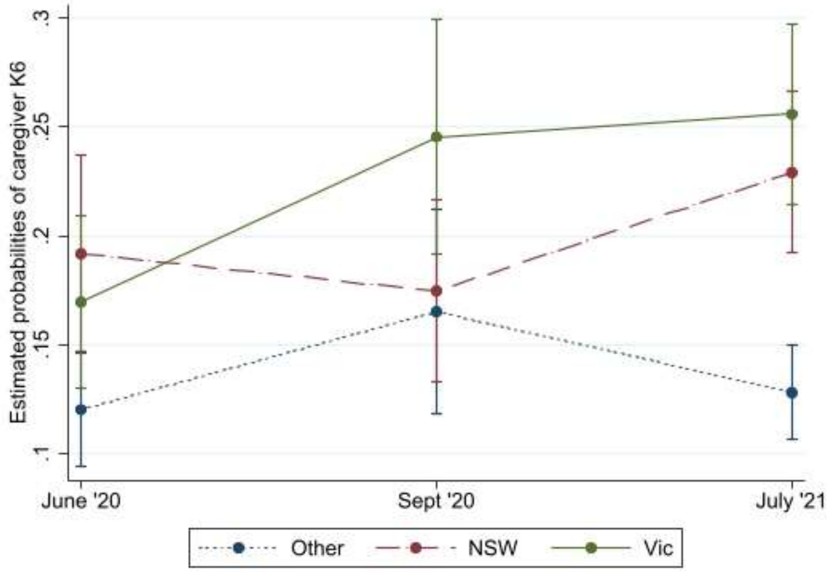
Estimated probabilities of caregiver K6
.1
.15
.2
.25
.3
June '20
Sept '20
July '21
Other
NSW
Vic

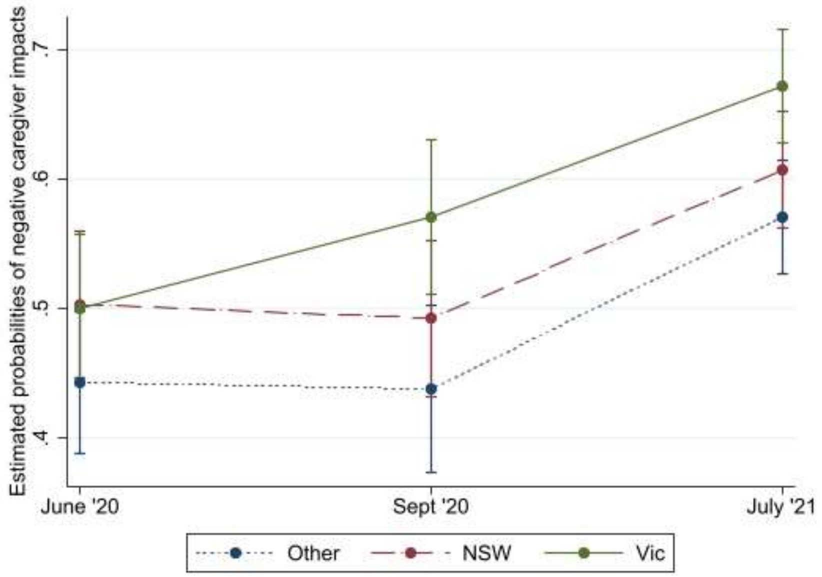
Estimated probabilities of negative caregiver impacts
.4
.5
.6
.7
June '20
Sept '20
July '21
Other
NSW
Vic

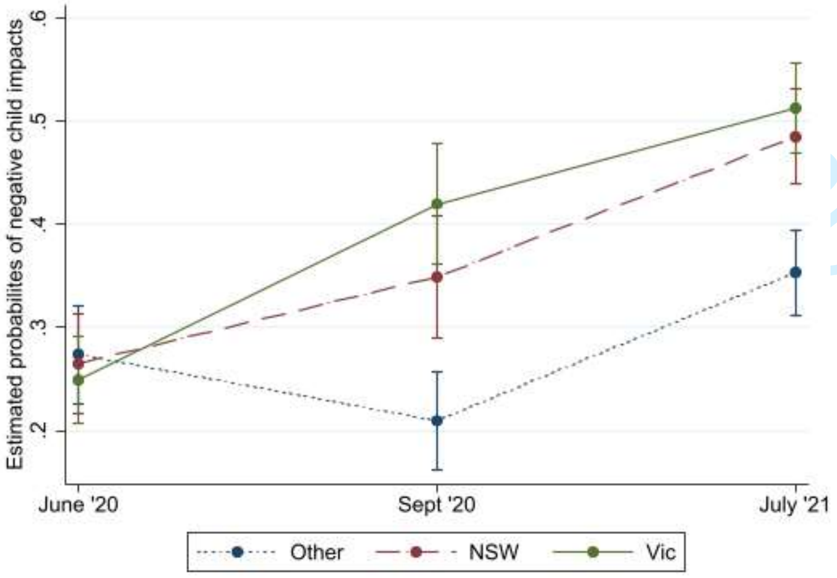
Estimated probabilites of negative child impacts
.2
.3
.4
.5
.6
June '20
Sept '20
July '21
Other
NSW
Vic

Supplementary Table 1: Estimated probabilities over time (by survey) and by state/territory (as a proxy for total length of lockdown) for the three mental health measures (graphed in Figure 1)

A. Poor caregiver mental health (Kessler-6)

	Proportion[a, b]	Risk Difference[a, b]	95% Confidence Interval[a, b]		p-values[a, b]
			Lower	Upper	
**Other States + W1 June 2020**	0.12				
**Other States + W2 Sep 2020**	0.17	0.05	-0.01	0.10	0.093
**Other States + W3 July 2021**	0.13	0.01	-0.02	0.04	0.589
**NSW + W1 June 2020**	0.19	0.07	0.02	0.12	0.005
**NSW + W2 Sep 2020**	0.17	0.05	0.01	0.10	0.024
**NSW + W3 July 2021**	0.23	0.11	0.06	0.15	0.000
**VIC + W1 June 2020**	0.17	0.05	0.00	0.10	0.033
**VIC + W2 Sep 2020**	0.25	0.13	0.07	0.18	0.000
**VIC + W3 July 2021**	0.26	0.14	0.09	0.18	0.000

[a] All estimates are adjusted for caregiver's gender, family structure (sole-caregiving), parent's education, Socio-Economic Indexes for Areas Index of Relative Disadvantage (SEIFA) score, and cultural and linguistic diversity as well as weighted using national demographic distributions for caregiver age, gender, family structure (sole-caregiving, number of children and any under 5 years), state/territory and SEIFA.
[b] Generalised linear model of the binomial family was used for analysis.

Confidential: For Review Only

B. Perceived negative impacts of the pandemic on caregiver mental health

	Proportion[a, b]	Risk Difference[a, b]	95% Confidence Interval[a, b]		p-values[a, b]
			Lower	Upper	
**Other States + W1 June 2020**	0.44				
**Other States + W2 Sep 2020**	0.44	0.00	-0.09	0.08	0.912
**Other States + W3 July 2021**	0.57	0.12	0.05	0.19	0.000
**NSW + W1 June 2020**	0.50	0.06	-0.02	0.14	0.132
**NSW + W2 Sep 2020**	0.49	0.05	-0.03	0.13	0.224
**NSW + W3 July 2021**	0.61	0.16	0.09	0.23	0.000
**VIC + W1 June 2020**	0.50	0.06	-0.02	0.14	0.130
**VIC + W2 Sep 2020**	0.57	0.13	0.05	0.21	0.001
**VIC + W3 July 2021**	0.67	0.23	0.16	0.30	0.000

[a] All estimates are adjusted for caregiver's gender, family structure (sole-caregiving), parent's education, Socio-Economic Indexes for Areas Index of Relative Disadvantage (SEIFA) score, and cultural and linguistic diversity as well as weighted using national demographic distributions for caregiver age, gender, family structure (sole-caregiving, number of children and any under 5 years), state/territory and SEIFA.

[b] Generalised linear model of the binomial family was used for analysis.

Confidential: For Review Only

C. Perceived negative impacts of the pandemic on children's mental health

	Proportion[a, b]	Risk Difference[a, b]	95% Confidence Interval[a, b]		p-values[a, b]
			Lower	Upper	
**Other States + W1 June 2020**	0.27				
**Other States + W2 Sep 2020**	0.21	-0.07	-0.13	-0.00	0.046
**Other States + W3 July 2021**	0.35	0.08	0.02	0.14	0.014
**NSW + W1 June 2020**	0.27	-0.01	-0.08	0.06	0.826
**NSW + W2 Sep 2020**	0.35	0.08	0.00	0.15	0.049
**NSW + W3 July 2021**	0.48	0.21	0.15	0.28	0.000
**VIC + W1 June 2020**	0.25	-0.02	-0.08	0.04	0.527
**VIC + W2 Sep 2020**	0.42	0.15	0.07	0.22	0.000
**VIC + W3 July 2021**	0.51	0.24	0.18	0.30	0.000

[a] All estimates are adjusted for caregiver's gender, family structure (sole-caregiving), parent's education, Socio-Economic Indexes for Areas Index of Relative Disadvantage (SEIFA) score, cultural and linguistic diversity, parent's mental health (Kessler-6) and clustering at the level of family as well as weighted using national demographic distributions for caregiver age, gender, family structure (sole-caregiving, number of children and any under 5 years), state/territory and SEIFA.
[b] Generalised linear model of the binomial family was used for analysis.

Confidential: For Review Only
